# Transcriptome and Small RNA Profiling of Potato Virus Y Infected Potato Cultivars, Including Systemically Infected Russet Burbank

**DOI:** 10.3390/v14030523

**Published:** 2022-03-03

**Authors:** Brian T. Ross, Nina Zidack, Rose McDonald, Michelle L. Flenniken

**Affiliations:** 1 Department of Plant Sciences and Plant Pathology, Montana State University, Bozeman, MT 59717, USA; brian.ross6@student.montana.edu (B.T.R.); rosemmcdonald812@gmail.com (R.M.); 2 Montana State Seed Potato Certification Lab, Department of Plant Sciences and Plant Pathology, Montana State University, Bozeman, MT 59717, USA; nzidack@montana.edu

**Keywords:** Potato virus Y, small RNAs, alternative splicing, extreme resistance, tolerance

## Abstract

Potatoes are the world’s most produced non-grain crops and an important food source for billions of people. Potatoes are susceptible to numerous pathogens that reduce yield, including Potato virus Y (PVY). Genetic resistance to PVY is a sustainable way to limit yield and quality losses due to PVY infection. Potato cultivars vary in their susceptibility to PVY and include susceptible varieties such as Russet Burbank, and resistant varieties such as Payette Russet. Although the loci and genes associated with PVY-resistance have been identified, the genes and mechanisms involved in limiting PVY during the development of systemic infections have yet to be fully elucidated. To increase our understanding of PVY infection, potato antiviral responses, and resistance, we utilized RNA sequencing to characterize the transcriptomes of two potato cultivars. Since transcriptional responses associated with the extreme resistance response occur soon after PVY contact, we analyzed the transcriptome and small RNA profile of both the PVY-resistant Payette Russet cultivar and PVY-susceptible Russet Burbank cultivar 24 hours post-inoculation. While hundreds of genes, including terpene synthase and protein kinase encoding genes, exhibited increased expression, the majority, including numerous genes involved in plant pathogen interactions, were downregulated. To gain greater understanding of the transcriptional changes that occur during the development of systemic PVY-infection, we analyzed Russet Burbank leaf samples one week and four weeks post-inoculation and identified similarities and differences, including higher expression of genes involved in chloroplast function, photosynthesis, and secondary metabolite production, and lower expression of defense response genes at those time points. Small RNA sequencing identified different populations of 21- and 24-nucleotide RNAs and revealed that the miRNA profiles in PVY-infected Russet Burbank plants were similar to those observed in other PVY-tolerant cultivars and that during systemic infection ~32% of the NLR-type disease resistance genes were targeted by 21-nt small RNAs. Analysis of alternative splicing in PVY-infected potato plants identified splice variants of several hundred genes, including isoforms that were more dominant in PVY-infected plants. The description of the PVY^N-Wi^-associated transcriptome and small RNA profiles of two potato cultivars described herein adds to the body of knowledge regarding differential outcomes of infection for specific PVY strain and host cultivar pairs, which will help further understanding of the mechanisms governing genetic resistance and/or virus-limiting responses in potato plants.

## 1. Introduction

Potato (*Solanum tuberosum*) is the third most important food crop, in terms of worldwide production, with nearly 400 million metric tons produced per year [1]. Potato cultivation and consumption from 1990–2020 increased steadily in China, India, and sub-Saharan Africa. The global human population is projected to grow by two billion by the year 2050 with the most growth occurring in developing countries [2]. Balancing the need to feed the world’s population while preserving biodiversity will be a challenge. Planting crops that are more resistant to pathogens and abiotic stressors and, therefore, require fewer inputs is one way to minimize the impact of agriculture. Potatoes are reliably grown in diverse landscapes and environmental conditions, but abiotic and biotic stressors, including water stress, heat stress, and pathogens can negatively impact yield and tuber quality [3]. Reliance upon clonal propagation renders potato production more vulnerable to vertical pathogen transmission and spread as compared to crops planted with true seed [4].

Strains of the Potyvirus, Potato virus Y (PVY), are the most damaging potato-infecting viruses worldwide (reviewed in [4,5]). PVY infection can render tubers unmarketable and decrease yield [6]. PVY is a positive sense, single-stranded RNA (+ssRNA) virus that infects many Solanaceous plant species, including the economically important vegetables potato, tomato, and pepper [7]. PVY infections of potato plants and tubers arise from non-circulative, stylet-borne aphid transmission, mechanical transmission in field settings, and from planting infected tubers [8,9]. Potato plants that are infected with PVY can display a wide range of symptoms and the severity of the infection depends on the combination of the PVY strain, potato cultivar, and the environmental conditions [10,11,12] (reviewed in [13]). Plants with extreme resistance to viruses rarely exhibit symptoms and have little to no detectable virus replication or spread beyond the infection site, whereas susceptible cultivars may have mosaic and/or crinkled leaves and/or exhibit necrosis in either leaves and/or tubers and develop systemic infections [4]. PVY-incidence in the field can increase greater than 10-fold from the start of the growing season to harvest, and a yield reduction of 150 lbs/acre is estimated for every 1% increase in PVY prevalence [14]. Pathogen-free seed and genetic resistance to PVY are the most economical and environmentally sustainable ways to stem losses and the spread of PVY.

Like many other RNA viruses, genetic variation in PVY is generated via error-prone replication and recombination [15]. Recombination events between the once prevalent PVY^O^ and PVY^N^ strains has given rise to many recombinant strains, including PVY^NTN^ and PVY^N-Wi^, which are now the most globally common and economically destructive [16]. These recombinant PVY strains are more prevalent than the parent strains largely due to their ability to evade detection and replicate in potato cultivars with strain-specific resistance. The rise of destructive recombinant strains may be, in part, due to selection pressures imposed by resistant cultivars, as increased local prevalence of recombinant strains strongly correlates with increased propagation of cultivars with strain-specific resistance [11].

Cultivar-specific PVY-resistance is largely dependent on host protein recognition of a viral effector protein [17]. This response, termed effector triggered immunity (ETI), is typified by the build-up of reactive oxygen species (ROS) in infected and surrounding cells and the induction of defense related proteins (e.g., pathogenesis-related (PR) proteins). The hypersensitive response (HR) can be induced by ETI and results in programmed cell death within the infected and surrounding areas after ROS accumulation (reviewed in [18,19]). HR prevents or limits the spread of pathogens that require living tissue to survive and replicate. ETI also results in the accumulation of the key immune signal, salicylic acid (SA), first in the infected area and then systemically, thereby priming non-infected distant tissues for possible pathogen invasion [20]. Planting of PVY-resistant potato cultivars limits growers’ losses but also results in the evolution and selection of virus strains that produce effector proteins with slightly altered recognition motifs. For example, the potato *Ny_tbr_* gene confers resistance to PVY^O^ by recognizing and binding two amino acid motifs (IGN and CCCT) on the PVY-encoded suppressor of RNA silencing, helper component protease (HC-Pro) [21]. The host plant does not recognize PVY^N^ because the locations of the HC-Pro recognition motifs are reversed relative to PVY^O^. Similarly, recombinant PVY-strains often encode HC-Pro proteins with unrecognizable structural conformations, and, in turn, these strains escape host detection and successfully replicate and spread [21,22].

Immune functions in all organisms must be finely regulated. In plants, many forms of pathogen resistance (e.g., ETI and HR) are controlled by a suite of nucleotide binding, leucine-rich repeat proteins (NLRs) [18]. NLR proteins are responsible for detecting intracellular pathogens and initiating an appropriate immune response. NLR abundance and function are regulated at the transcriptional, post-transcriptional, and translational levels [23]. The abundance of NLR transcripts increases after pathogen detection and may heighten detection and defense responses in surrounding cells [24]. Slight changes of NLR transcript levels (e.g., less than two-fold) in the absence of pathogens can result in constitutively activated immune responses and growth defects in Arabidopsis [25,26].

RNA interference (RNAi) mechanisms, including the small interfering RNA (siRNA) and microRNA (miRNA) pathways, are critical for antiviral defense and the regulation of gene expression in plants [27]. In brief, siRNAs are produced by Dicer-like (DCL) proteins that cleave cytosolically located long double-stranded RNAs (dsRNAs), including the replicative intermediate form of +ssRNA viruses, into 21–24 nucleotide (nt) siRNA duplexes [28]. An ARGONAUTE (AGO) protein in complex with other cofactors retains one strand of the siRNA duplex to form the RNAi induced silencing complex (RISC) [29]. The guide siRNAs facilitate binding of the RISC to cognate target RNA sequences, which are either sliced or translationally inhibited [30,31,32].

MicroRNAs primarily target host transcripts to regulate mRNA levels in the cell and are important in modulating potato gene expression, including genes involved in immune responses [33]. MicroRNAs are 21–22-nt small RNAs produced from DCL-mediated cleavage of host-encoded dsRNA transcripts. The production of phased, small interfering RNAs (phasiRNAs) occurs when one or two miRNA-loaded RISC complexes bind to a single RNA transcript and cleave it [34]. RNA-dependent RNA polymerase 6 (RDR6) is recruited to the cleaved transcript which is used as a template for dsRNA synthesis. The long dsRNA is cleaved by DCL every 21–22 nucleotides, resulting in a characteristic ‘phasing’ pattern [35]. The resulting phasiRNAs are incorporated into RISC where they target cognate sequences for silencing and effectively amplify the miRNA signal. MicroRNAs and phasiRNAs are key post-transcriptional regulators of NLR gene expression [24]. For example, miR482 and miR2118 families in *Solanaceae* target conserved P-loop domains within NLR transcripts to initiate phasiRNA biogenesis, which results in reduced expression of target genes [36]. The enzymatic activity of NLRs is also regulated post-translationally (reviewed in [19]). Proteins bound to ATP recognize and bind pathogen effectors and elicit an immune response whereas ADP-bound proteins do not [37]. It is hypothesized that NLRs likely exist in either an ATP or ADP-bound state in the cell [38]. According to this model, the population of NLRs in a cell is constantly in equilibrium between these “On” and “Off” states, with the equilibrium shifted towards the ADP-bound, “Off” state until pathogen recognition and ligand binding [38].

To better understand potato antiviral defense mechanisms, we investigated PVY-infection in two potato cultivars: Russet Burbank and Payette Russet, and identified the transcriptional level changes and differences in small RNA profiles at a single early time point in both cultivars and during the development of systemic infection of Russet Burbank plants. Russet Burbank is the most widely grown cultivar in the United States, due primarily to its uniform tuber production and favorable cooking qualities [39]. Russet Burbank is susceptible to many pathogens, including PVY. Previous studies examined the transcriptional responses of Russet Burbank plants infected with two PVY strains (i.e., PVY^O^, PVY^NTN^) 4- and 10-hours post-infection (hpi) in inoculated leaves, and other studies characterized large transcriptional changes in PVY-infected plants 1- and 3-days post-infection [12,13]. In our previous studies, we determined that systemic PVY^N-Wi^ infection of Russet Burbank takes a few weeks to develop [40], therefore, in this study we evaluated the transcriptional and small RNA responses early in infection (i.e., 24 hpi in inoculated leaves), as well as later time points (i.e., 1 and 4 weeks post-infection (wpi)) in upper uninoculated leaves to identify the changes during development of systemic PVY-infection.

Resistance to PVY is usually virus strain-dependent and immune activation in plants can present various visual symptoms [11,12,19,41,42] (reviewed in [4,13,19]). Extreme resistance is defined by a lack of symptoms and little to no detectable viral replication after inoculation. Payette Russet and other potato cultivars containing *Ry_sto_*-mediated resistance are resistant to most strains of PVY and Potato virus A (PVA) [43]. Payette Russet has ‘extreme resistance’ governed by the NLR gene, *Ry_sto_*, which has been introgressed from the wild potato species, *S. stoloniferum*, into commercial cultivars [43]. The *Ry_sto_* locus has been mapped to the distal arm of potato chromosome 12, which contains four clusters of resistance genes [44,45]. An NLR protein encoded by the *Ry_sto_* gene recognizes and binds to the coat protein encoded by strains of PVY and PVA [43]. The transcriptional responses following pathogen recognition by *Ry_sto_* that result in an extreme resistance phenotype are not completely understood. Therefore, in parallel with our experiments in Russet Burbank, we examined the transcriptional and small RNA responses mounted by the PVY-resistant cultivar Payette Russet 24 hours post PVY^N-Wi^inoculation.

We determined that PVY^N-Wi^ infection resulted in broad changes to the transcriptional and small RNA profiles. Specifically, thousands of genes, many of which may be involved in PVY-resistance, were differentially expressed in Payette Russet, including a leucine-rich repeat transmembrane protein kinase and genes involved in terpene synthesis. Similarly, PVY^N-Wi^ infection of Russet Burbank plants also resulted in thousands of differentially expressed genes, including greater expression of genes that encode protease inhibitors, heat shock proteins, and a terpene synthase. In addition, we characterized PVY-associated changes in host small RNA profiles which had not been described in upper, uninoculated leaves at the early stages of systemic infection and an analysis of alternative splicing in response to PVY infection, which has also not been previously described. Overall, this study improves our understanding of the transcriptional and post-transcriptional responses to PVY infection in potato and identifies genes that may be of interest to future studies.

## 2. Materials and Methods

### 2.1. Potato Sources and Growing Conditions

Potato cultivars ‘Russet Burbank’ and ‘Payette Russet’ were propagated as pre-nuclear tissue culture plantlets using 4.44 g/L Murashige and Skoog medium with Gamborg’s vitamins (PhytoTechnology Laboratories, Shawnee Mission, KS, USA). After five weeks, plantlets were transplanted into 5″ pots containing Sunshine Mix 1 (Sungro Horticulture, Vancouver, BC, Canada) and grown in a growth chamber at 22 °C under a 16:8 day:night photoperiod. Watering was performed as needed and MiracleGro Shake ‘n Feed All Purpose Plant food was applied biweekly. Plants were grown for four weeks in pots prior to mechanical inoculation.

### 2.2. PVY Inoculum Preparation and Inoculation Protocol

**Figure 1:**
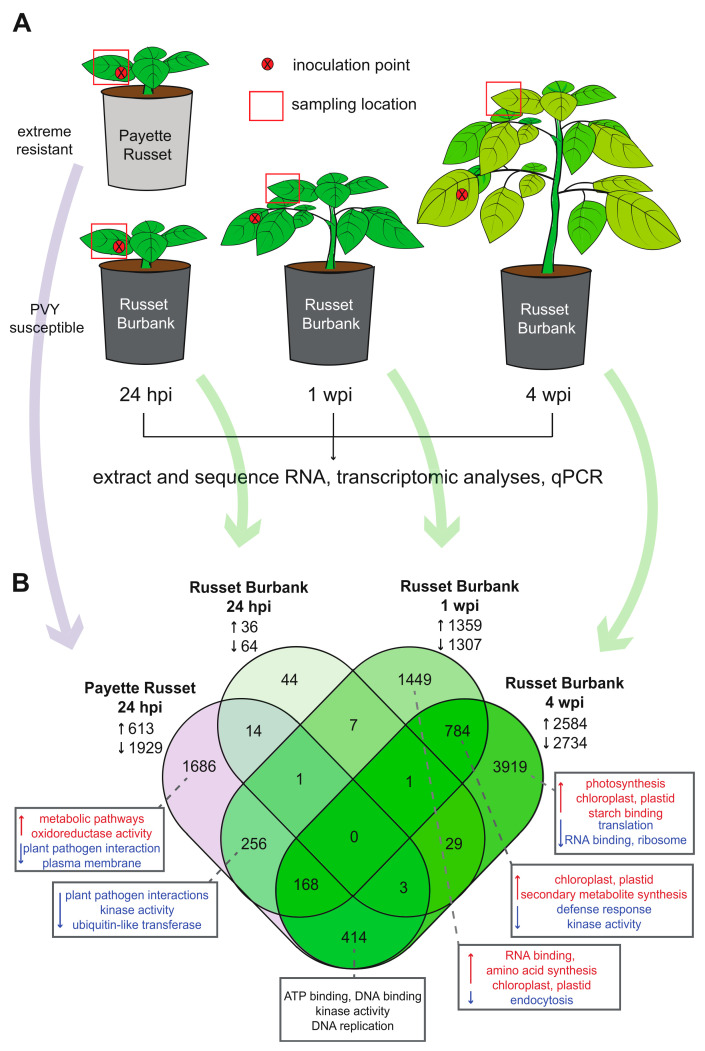
Transcriptome level evaluation of the response of two potato cultivars indicates that response to Potato virus Y-inoculation varies by cultivar and time point. (**A**) Schematic representation of the experiments performed to investigate potato antiviral immune responses. Potato plants were infected with PVY or mock-infected (*n* = 6 plants per treatment, per time point, per biological replicate). Leaf tissue samples were collected from the inoculation point at 24 hours post-infection (hpi) for both cultivars and at 1- and 4-weeks post-infection (wpi) in PVY-susceptible Russet Burbank plants. RNA was extracted from leaf tissue samples and prepared for either qPCR or high throughput sequence analysis. qPCR was used to quantify PVY abundance in all plants and to validate differential gene expression results for a subset of genes in a subset of plants from all three biological replicates. (**B**) Potato transcriptional responses to PVY-infection are time- and cultivar-dependent. The Venn diagram illustrates the number of unique and shared differentially expressed genes (DEGs) in Payette Russet (blue) and Russet Burbank (shades of green) plants in response to PVY infection. No differentially expressed genes were shared between all time points and cultivars, though hundreds of differentially expressed genes were shared between Payette Russet at 24 hpi and Russet Burbank at 1 and 4 wpi. Full lists of differentially expressed genes and their corresponding fold changes are included in Supplemental File S1.

The PVY strain Wilga (PVY^N-Wi^) (NCBI Accession: HQ912863) was obtained from the Karasev Lab at the University of Idaho. PVY^N-Wi^ stock was maintained in tobacco plants (*Nicotiana tabacum* ‘Samsun’), which were cultivated from seed with a 24-hour (h) photoperiod at 22 °C in 10″ pots, as previously described [40]. The genome sequence of PVY^N-Wi^ utilized in this study was verified by RNASeq and shares 99.7% nucleotide identity with the PVY^N-Wi^ reference genome (HQ912863). Virus inoculum was prepared by placing 1 g infected tobacco leaf tissue in 10 mL of inoculation buffer (0.05 M disodium phosphate, 0.05 M monopotassium phosphate, pH 7.5) in a 12 cm by 12 cm universal extraction bag (Bioreba, Reinach, Switzerland). Leaf tissue was homogenized in buffer using a tissue homogenizer (Bioreba, Reinach, Switzerland) mounted on a drill press (Craftsman, Hoffman Estates, IL, USA). Carborundum powder was sprayed onto a targeted potato plant leaf (i.e., the fourth leaf down from the top) and a cotton-tipped swab was used to gently abrade the leaf surface. PVY inoculum (200 μL) was applied to the leaf surface and spread across the abraded area with a pipette tip. Leaves were rinsed with water 1 h after inoculations to remove any residual buffer. Mock-infected plants were treated with inoculation buffer only, with all other conditions kept constant. Sampling of leaf tissue occurred 24 hpi, and then at weekly intervals up until 4 wpi. Approximately 200 mg of leaf tissue was taken from the tip of the inoculated leaf at 24 hpi. Approximately 200 mg of leaf tissue was taken from upper, uninoculated leaves (the fourth leaf down from the apical meristem) from 1–4 wpi. Leaf tissue was stored in a −80 °C freezer until analysis. For each of three biological replicates of the experiment, six plants were mechanically inoculated with PVY^N-Wi^ (NCBI Accession: HQ912863) or inoculation buffer (i.e., mock-infected) [40]. RNA sequencing performed on three of the six plants per treatment group in biological replicate #1 (i.e., *n* = 3 RNA samples per treatment group per time point), and qPCR was performed on all the plant samples in this study (i.e., *n* = 6 plants per treatment group, sampled at three time points post-inoculation) for each biological replicate of the experiment and all three biological replicates performed as part of this study (Figure 1, Supplementary File S1A).

### 2.3. RNA Isolation and cDNA Synthesis

RNA was extracted from leaf tissue samples using TRIzol reagent (Life Technologies, Carlsbad, CA, USA) according to the manufacturer’s instructions. Reverse transcription reactions (25 μL) were performed using 2 μg of total RNA and random hexamer primers (500 ng) (IDT, Coralville, IA, USA) incubated with Maloney murine leukemia virus (M-MLV) reverse transcriptase (Promega, Madison, WI, USA) for 1 h at 37 °C, according to the manufacturer’s instructions.

### 2.4. Quantitative PCR (qPCR)

Quantitative PCR was performed in triplicate using a CFX Connect Real Time machine (BioRad, Hercules, CA, USA). Each 20 μL reaction contained 2 μL of cDNA, 1× ChoiceTaq Mastermix (Denville, Holliston, MA, USA), 0.1 μM of each forward and reverse primer, 1× SYBR Green (Life Technologies, Carlsbad, CA, USA), and 25 mM MgCl_2_. To estimate the relative PVY abundance based on a standard curve, the corresponding segments of PVY were cloned into plasmids. Plasmid standards containing from 10^3^ to 10^9^ copies per reaction were used as templates for qPCR to generate standard curves. The accurate detection limit was 10^3^ copies per reaction for the PVY^N-Wi^ qPCR primer set. The linear standard equations for the PVY^N-Wi^ qPCR primer set, generated by plotting the crossing point (Cp) versus the log_10_ of the initial plasmid copy number was as follows: PVY^N-Wi^ Cp = −3.20x + 44.54, *R*^2^ = 0.99. Quantitative PCR reactions containing no template served as negative controls. Melt point analyses, agarose gel electrophoresis, and sequencing of initial qPCR products were used to verify qPCR specificity.

The ΔΔC(t) method was used to calculate the relative abundance of the gene of interest (GOI) in individual leaf tissue samples because it was most accurate; the ΔΔC(t) method ensures that results are not skewed by inadvertent differences in RNA reverse-transcription efficiencies and starting cDNA template abundance. The ΔC(t) for each sample was calculated by subtracting the C(t) of the reference gene *Exosome-associated protein* from the GOI C(t). The potato gene encoding *Exosome-associated protein* was selected as an appropriate housekeeping gene for qPCR because analysis of the transcriptomic data presented herein confirmed that *Exosome-associated protein* expression levels were similar in all sequenced libraries. The ΔΔC(t) was calculated by subtracting the average virus-infected ΔC(t) values from the ΔC(t) values for each treatment group. For host gene expression analyses and transcriptomic analysis validation, the percent gene expression for each gene of interest (GOI) was calculated using the following formula: 2^−ΔΔC(t)^ × 100 = % gene expression, in which ΔC(t) = GOI C(t) − *Ref* C(t), and ΔΔC(t) = sample ΔC(t)−mock-infected control ΔC(t) [46]. Wilcoxon rank sum tests were used to identify statistical differences in host gene expression.

### 2.5. Transcriptome Library Preparation and Sequencing

Prior to transcriptome library preparation, RNA from each sample was DNase treated using an on-column DNase Treatment (Qiagen). The RNA quality was assessed using an Agilent 2200 Bioanalyzer (Santa Clara, CA, USA) and quantified with a Thermo Scientific NanoDrop 2000 Spectrophotometer (Waltham, MA, USA). The RNA was sent to the Roy J. Carver Biotechnology Center at the University of Illinois at Urbana–Champaign for library preparation (Illumina TruSeq Stranded RNA Sample Prep kit). mRNA libraries were poly-A purified and sequenced on two 2 × 100 nucleotide lanes of the Illumina HiSeq 4000 which produced over 1.2 billion reads (15.8× coverage over the ~840 Mb pair potato genome). Small RNA libraries were sequenced on one 1 × 50 nucleotide lane of the Illumina HiSeq 4000 which produced over 370 million reads. Sequence data was deposited into the NCBI Sequence Read Archive under submission number PRJNA768797. The analyzed sequence data is included in Supplemental Files S1–S4.

### 2.6. Differential Gene Expression Analysis

FastQC was used to remove low quality reads (<Q30). Illumina adapters were trimmed with Trimmomatic, and reads were aligned to the *S. tuberosum* DM v6.1 genome assembly with Hisat2; on average, ~85% of reads from each sample were mapped to the genome (Supplementary File S1B). Alignments were assembled and count indices were made for each library via Stringtie [47]. Differential expression analysis of alignments was performed with the DESeq2 in R. Genes with strong evidence of differential expression between treatments (adj. *p*-value ≤ 0.05) were further analyzed with the gene and functional ontology program, g:Profiler [48]. Small RNAs were aligned to the potato reference genome (DM v6.1) with Shortstack [49]. PhasiRNAs were identified from small RNA clusters with phased scores > 30. MicroRNAs detailed in the text were identified as putative miRNAs by Shortstack and aligned using BLAST (basic local alignment search tool) with experimentally verified miRNAs using Penn State’s Small RNA database server, which facilitates searches against miRbase as well as small RNAs identified by Shortstack from hundreds of plant reference libraries [50,51]. Differential expression of small RNAs was calculated by taking the raw counts data from Shortstack and inputting those data into DESeq2 version 1.33.4 [47].

### 2.7. Blast Search of Potato Transcript Sequences against Potato Genome, Wild Potato Species, and All Organisms

The Spuddb and NCBI NR sequence, annotation, accession, and taxonomy sources, and the DIAMOND executable were downloaded on 14 September 2021.

The DIAMOND database was built, and DIAMOND blastx was run on r5d.8xlarge and c5ad.24xlarge AWS EC2 instances, respectively, using the Amazon Linux 2 AMI as the machine image. Simplified versions of the commands building the DIAMOND database of NCBI’s NR database and performing the DIAMOND blastx search comparing SpudDB gene models and NCBI’s NR were as follows: “./diamond makedb --in nr.gz--taxonmap prot.accession2taxid.FULL.gz --taxonnodes./taxdmp/nodes.dmp --taxonnames./taxdmp/names.dmp --db nr.full”; “./diamond blastx –query DM_1-3_516_R44_potato.v6.1.working_models.cdna.fa --db nr.full --sensitive --iterate --evalue 0.001 --max-target-seqs 200 --max-hsps 1 --index-chunks 1 --block-size 7 --unal 1 --log --outfmt 6 qseqid sseqid evalue bitscore score salltitles -o diamond_results.tsv”.

A custom script in R was used to extract the best (first) hit for each of *S. tuberosum*, potato breeding species, and ‘any organisms’ using regular expressions on the salltitles field. The potato breeding species used for these extractions were: *S. tuberosum*, *S. acaule*, *S. bulbocastanum*, *S. candolleanum*, *S. commersonii*, *S. demissum*, *S. maglia*, *S. microdontum*, *S. stoloniferum*, and *S. vernei*. The results can be found in Supplemental File S1C and Supplemental File S4.

## 3. Results and Discussion

### 3.1. Transcriptome Level Assessment of Potato Virus Y (PVY)-Infected Potato Cultivars

To characterize the transcriptional responses of PVY-susceptible Russet Burbank plants during development of systemic infection, plants were sampled at three time points after infection (24 hpi, 1 wpi, and 4 wpi) with the inoculated leaf being sampled at 24 hpi, and upper, uninoculated leaves sampled at the later time points. In addition, a single time point early post PVY^N-Wi^ inoculation (i.e., 24 hpi) was obtained from PVY-resistant Payette Russet plants, in order to examine responses associated with resistance, as well as to compare responses between the two cultivars. At each time point post-infection, leaf tissue samples were obtained from six plants per treatment (i.e., PVY- or mock-infected) and RNA was isolated and quantified (Figure 1A). Virus abundance in individual plants at each time point were quantified by quantitative PCR (qPCR) using RNA copy number (i.e., viral genomes and transcripts) as a proxy for virus abundance (Supplemental File S1A). Transcriptome sequencing libraries were produced with RNA obtained from a subset of individual plants in all treatment groups in biological replicate #1. High throughput sequencing was carried out using an Illumina HiSeq 4000. The resulting reads were aligned with HiSat2, and differential expression was assessed using Stringtie and DESeq2 [47,52]. The reads were aligned to the potato DM version 6.1 genome [53] (Supplemental File S1B). Transcript sequences from the DM version 6.1 working gene models were BLAST searched against potato, wild potato species commonly used for breeding purposes, and all organisms to provide a link between the potato genome online resources and the NCBI online resources (Supplemental File S1C–K).

**Figure 2:**
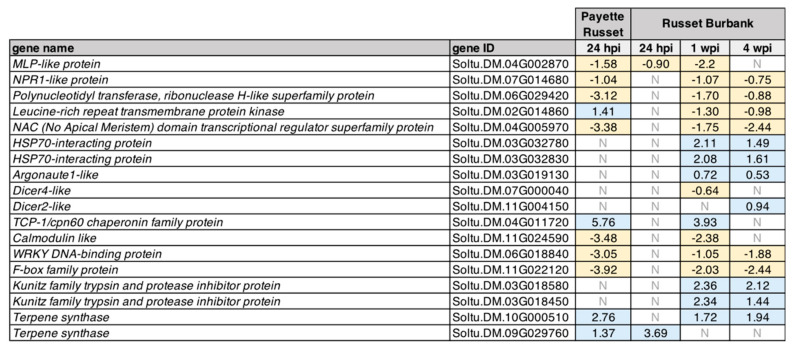
Select genes that exhibited notable changes in expression in PVY-infected potato plants relative to mock-inoculated plants. Table highlights a small subset of DEGs identified by transcriptomic analysis; the relative expression of these genes of interest varies by time point (24 hpi, 1 and 4 wpi) and potato cultivar (Payette Russet and Russet Burbank). Highlighting indicates lower (yellow) or greater (blue) expression in virus-infected plants as compared to mock-infected control plants and “N” is used for non-detectable and/or non-statistically significant values.

Transcriptome level analyses (RNAseq) identified thousands of potato genes that exhibited differential expression within the context of PVY infection, potato cultivar, and time point post-infection (Figure 1 and Figure 2, and Supplemental File S1). Notable genes include those involved in plant immunity, including RNAi (i.e., *Dicer2-like*, *Dicer4-like, Argonaute1-like*) and plant immune signaling (i.e., *NPR1-like*, *WRKY DNA-binding protein*), which exhibited reduced expression at the time points we evaluated, as well as other genes potentially involved in plant immunity (i.e., *Kunitz family trypsin and protease inhibitor protein*, *HSP70-interacting protein*) that exhibited greater expression (Figure 2). For example, expression of the salicylic acid regulator, *NPR1-like*, was reduced in PVY-infected Payette Russet plants at 24 hpi (−1.04 log_2_ fold change), and in PVY-infected Russet Burbank plants at 1 wpi (−1.07 log_2_ fold change) and 4 wpi (−0.75 log_2_ fold change), compared to the relevant mock-infected plants (Figure 2). These results may indicate a decrease in salicylic acid signaling at these time points. Salicylic acid signaling is an important component of *Ny-1* gene-mediated PVY resistance [42] and, therefore, reduced expression of *NPR-1-like* at the assessed time points may indicate that SA-mediated responses and signaling were activated earlier and the down-regulation represents an over-correction in the process of returning to homeostasis, or it is possible that PVY^N-Wi^ infection reduces the expression of this gene in order to facilitate virus replication and spreading.

**Figure 3:**
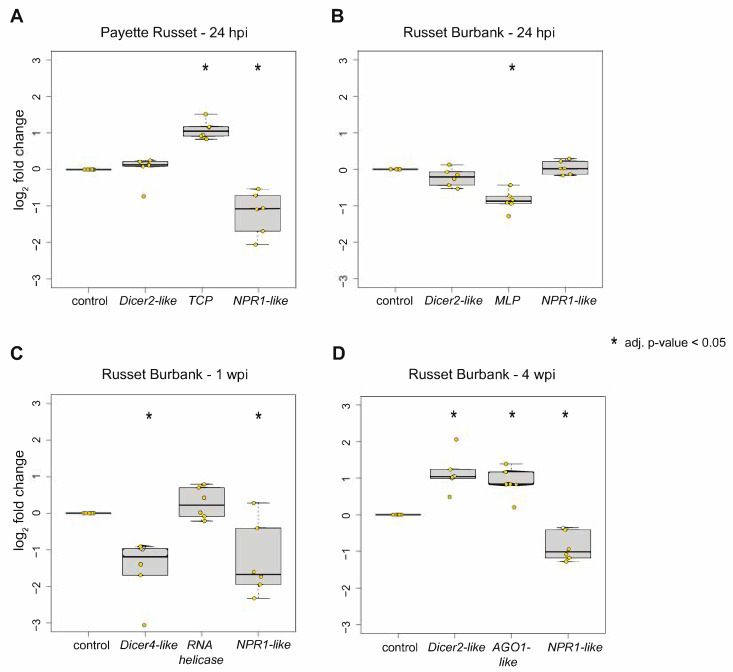
Potato transcriptome data supported by quantitative PCR analysis. Quantitative PCR (qPCR) was used to validate the differential expression analysis results for a subset of genes for each time point (24 hours post-inoculation, 1- and 4- weeks post-inoculation) and cultivar (PVY-resistant Payette Russet, PVY-susceptible Russet Burbank) used in this study. (**A**) qPCR assessment of the relative expression of *Dicer2-like* (Soltu.DM.11G004150), *TCP* (Soltu.DM.04G011720), and *NPR1-like* (Soltu.DM.07G014680) in PVY-inoculated Payette Russet leaves at 24 hpi relative level in mock-infected control plants, determined that their expression was similar (0.04 log_2_ fold change), higher (1.10 log_2_ fold change), and lower (−1.11 log_2_ fold change), respectively. (**B**) Evaluation of the expression of *Dicer2-like*, *MLP*, and *NPR1-like* in PVY-infected Russet Burbank plants at 24 hpi relative to uninfected controls indicated that only the expression of *MLP* (Soltu.DM.04G002870) was reduced by PVY-infection (−0.84 log_2_ fold change). (**C**) The qPCR gene expression results from upper, uninoculated leaves of PVY-infected Russet Burbank plants at 1 wpi compared to mock-infected controls indicate a reduced expression of *Dicer4-like* (Soltu.DM.07G000040) (−1.35 log_2_ fold change) and *NPR1-like* (−0.98 log_2_ fold change), and a non-statistically significant increase in the expression of a *putative mitochondrial RNA helicase expression* (Soltu.DM.12G023910) (0.32 log_2_ fold change). (**D**) The qPCR gene expression results from upper, uninoculated leaf samples of PVY-infected Russet Burbank plants at 4 wpi compared to uninfected control plants indicated that the expression of *Dicer2-like* (1.22 log_2_ fold change) and *AGO1-like* (Soltu.DM.03G019130) (0.92 log_2_ fold change) was increased, whereas the *NPR1-like* expression (−0.83 log_2_ fold change) was reduced. Statistical differences in gene expression between mock-infected and PVY-infected potato plants (*n* = 6) were performed using Wilcoxon rank sum test, * *p* < 0.05. All of the qPCR results mirrored the transcriptome level assessment results, except that the increased expression of RNA helicase in PVY-infected Russet Burbank plants was not statistically significant by qPCR whereas it was in the transcriptome data. This figure includes data from one representative biological replicate and the results from all three biological replicates are included (Supplemental Figures S1 and S2).

To validate transcriptome data, the expression of a subset of the differentially expressed genes (DEGs) identified for each time point and cultivar was also assessed by qPCR. Statistical differences in gene expression between PVY-infected and mock-infected potato plant samples (*n* = 6 per biological replicate; *n* = 18 total plant samples per treatment group in all three biological replicates) were evaluated using the Wilcoxon rank sum test, * *p* < 0.05 (Figure 3, Supplemental Files S1 and S2). Specifically, qPCR assessment of the relative expression of *Dicer2-like*, *NPR1-like*, and *TCP* in PVY-inoculated Payette Russet leaves at 24 hpi relative level in mock-infected control plants, determined that their expression was similar, lower, and higher, respectively (Figure 3A). Evaluation of the expression of *Dicer2-like*, *MLP*, and *NPR-1*, as in PVY-infected Russet Burbank plants relative to uninfected controls, indicated that only the expression of *MLP* was reduced by PVY-infection (Figure 3B). The qPCR gene expression results from upper, uninoculated leaves of PVY-infected Russet Burbank plants at 1 wpi compared to mock-infected controls indicate a reduced expression of *Dicer4-like* and *NPR1-like*, and a non-statistically significant increase in the expression of a putative mitochondrial RNA helicase expression (Figure 3C). The qPCR gene expression results from upper, uninoculated leaf samples of PVY-infected Russet Burbank plants at 4 wpi compared to uninfected control plants indicated that the expression of *AGO1-like* and *Dicer2-like* was increased, whereas the *NPR1-like* expression was reduced (Figure 3D). All of the qPCR results mirrored the transcriptome level assessment results, except that the increased expression of RNA helicase in PVY-infected Russet Burbank plants was not statistically significant by qPCR. The qPCR data validated the high throughput sequencing data and confirmed that the results were robust, as they were similar in three independent biological replicates (Supplemental Figures S1 and S2). Sequencing data were also used to confirm that the PVY^N-Wi^ genome used in this study shares 99.7% nucleotide identity with the PVY^N-Wi^ reference genome (HQ912863) (Supplemental Figure S3).

To evaluate the cellular pathways and mechanisms that are regulated in response to virus infection, genes that were differentially expressed in PVY-infected plants, compared to mock-infected plants, were further analyzed using gene ontology (GO) and cellular pathway enrichment analyses. Specifically, the GO program, g:Profiler, was used with the Phureja DM1-3 PGSC protein identification schema to characterize differentially expressed genes by pathway, molecular function, and biological process [48] (Figure 1B). Select GO terms and DEGs associated with particular comparisons are described in greater detail below.

#### 3.1.1. Transcriptomic Differences between PVY-Resistant Payette Russet and Susceptible Russet Burbank Potato Cultivars 24-hours Post PVY-Inoculation

The Payette Russet potato cultivar exhibits extreme resistance to PVY. Extreme resistance is characterized by little or no virus replication at the site of infection and a lack of visual symptoms of either infection or an immune response (i.e., hypersensitive response) [19,54]. There are a small number of studies describing the transcriptional responses of particular potato cultivars to specific PVY-strains, and only one that also investigates a cultivar with extreme resistance against PVY (i.e., Sante) [41] and, thus, there is little precedent for selecting sampling time point(s) that would best represent the extreme resistance response. Previous research has indicated that transcriptional changes can occur within hours of inoculation with PVY, though other research indicates that resistance responses due to HR and possibly extreme resistance also occur days after virus inoculation [12,13,55,56,57]. We hypothesized that the PVY-limiting immune responses in Payette Russet would occur soon after virus inoculation. Therefore, to characterize these initial immune responses and to be able to compare them to the same time point in Russet Burbank, samples were obtained from the PVY-inoculated leaves of potato plants at 24 hpi.

Virus levels in the PVY-infected leaves were near the limit of qPCR detection and accounted for less than or equal to 1% of the total reads in each of the 24 hpi sequencing libraries (Supplemental File S1B). Differential gene expression analysis of Payette Russet leaf samples at 24 hpi identified over 2000 genes that were differentially expressed in PVY-inoculated plants as compared to mock-inoculated plants (adj. *p* ≤ 0.05) (Figure 1B, Supplemental File S1D). The majority (1929 genes) exhibited decreased expression while 613 exhibited increased expression (Figure 1B, Supplemental File S1D). GO analysis of the differentially expressed genes in Payette Russet at 24 hpi indicated that genes involved in ATP binding, kinase activity, and DNA binding were enriched among genes that exhibited decreases in expression (adj. *p* ≤ 0.05) (Supplemental File S1E). Because this cultivar is resistant to PVY infection, it is likely that these DEGs contribute to initial events in potato antiviral defense. We identified differentially expressed genes with characterized functions in plant antiviral defense. For example, *NPR1-like (NONEXPRESSOR OF PATHOGENESIS-RELATED 1-LIKE)* controls cellular salicylic acid concentrations, which is a key signaling molecular controlling responses to pathogen infection [58,59]. RNA sequencing results indicate a decrease in transcription of *NPR1-like* (log_2_ fold change = −1.04), and qPCR analyses confirmed this result (Figure 1B, Figure 2, Supplemental Figures S1 and S2). Increased expression of *NPR1-like* results in greater cellular salicylic acid concentrations, so the observed decrease is possibly indicative of a waning immune response [60,61]. In another example, pathogenesis-related proteins (PR proteins) are induced in leaf tissue following pathogen detection and can be used as immune response markers in plants [62]. Seven different PR proteins exhibited decreased expression in PVY-treated Payette Russet leaves as compared to mock-infected plants, potentially indicating down-regulation of immune response genes at 24 hpi (Supplemental File S1D). PR1 proteins, which are a part of a variety of defense responses in plants, contain a defense-related signaling domain and bind sterols, though their defense function(s) require further characterization [63]. Few studies have examined transcriptional level responses to PVY in resistant cultivars. These studies include a microarray-based study of PVY^NTN^-inoculated resistant cultivar Sante and sensitive cultivar Igor in which the majority of DEGs (i.e., 299 and 190, respectively) exhibited reduced expression at 0.5 and 12 hpi [41]. Likewise, PVY^O^ infection of PVY-resistant Premier Russet exhibited a greater number of genes with reduced expression at 4 hpi but not at 10 hpi [12], whereas the number of genes with reduced expression (*n* = 273) was similar to the number of genes with increased expression (*n* = 216) in susceptible Russet Burbank plants. In contrast, PVY^N-Wi^-inoculated resistant cultivar Rywal plants at 24 hpi had a greater number of genes that exhibited increased expression (*n* = 1568) compared to those that had lower expression (*n* = 678) [42]. Together, these studies illustrate that transcriptional responses vary with potato cultivar, PVY-strain, time post-infection, and sampling site, which is understandable given that all of these factors also impact the symptoms and outcome of PVY infections [12,13,64]. While these and other studies have begun to characterize the spectrum of responses potato cultivars mount against specific PVY-strains, additional studies are needed to better understand the molecular mechanisms that limit virus infections, with the goal of identifying additional genetic resistance strategies that may be incorporated in potato breeding programs.

Russet Burbank is susceptible to most PVY strains, including PVY^N-Wi^, and thus, we wanted to compare the transcriptional changes between extreme resistant Payette Russet and Russet Burbank. Ninety-nine genes were differentially expressed at 24 hpi in PVY-infected Russet Burbank plants as compared to mock-infected plants (adj. *p* ≤ 0.05) (Supplemental File S1F,G). The majority of the DEGs (64 out of 100) exhibited lower expression while 36 genes exhibited higher expression in PVY-infected plants compared to the mock-infected control plants (Figure 1B, Supplemental File S1G). Gene ontology analysis indicated that PVY-infected Russet Burbank plants exhibited enrichment for DEGs involved in transmembrane transport, photosynthesis (photosystem I, II), and metabolism at 24 hpi (adj. *p* ≤ 0.05). Among the DEGs with increased transcription, terpene synthase activity, ion homeostasis, and transmembrane transporter activity were enriched. Among the DEGs with decreased transcription, kinase activity, chlorophyll binding, and photosynthesis activities were enriched (Supplemental File S1H). Genes involved in photosynthesis were also enriched in PVY^O^-inoculated resistant Premier Russet plants [12] and exhibited greater expression in the PVY^NTN^-resistant Sante cultivar and PVY^NTN^-susceptible cultivar Igor plants at 0.5 hpi, and lower expression than noninoculated control plants, by 12 hpi [41].

Like Payette Russet, the genes that exhibited the greatest increases in expression at 24 hpi included genes involved in transmembrane transport and terpene synthesis. Among the most statistically supported genes were a cation exchanger (Soltu.DM.09G008230, log_2_ fold change = 2.31), a cyclic nucleotide-gated cation channel protein (Soltu.DM.10G002160, log_2_ fold change = 1.90), and a Calcium-binding EF-hand family protein (Soltu.DM.10G027990, log_2_ fold change = −1.20) (Supplemental File S1G). These three genes are all implicated in ion exchange, particularly calcium ion exchange, which may be a key component of the hypersensitive response [65]. Similarly, three genes encoding terpene synthases (Soltu.DM.09G029760, Soltu.DM.09G029770, Soltu.DM.09G029880) exhibited increased expression at 24 hpi in Russet Burbank and Payette Russet (Supplemental File S1I). Terpenes are volatile organic compounds produced and released by plants to combat herbivory and infection [66,67]. The expression of terpene synthesis-related genes is in part controlled by cellular jasmonic acid concentrations [68]. The jasmonic acid pathway can act antagonistically to the salicylic acid pathway, which contributes to antiviral defense [69,70]. That the same terpene synthase genes exhibited increased expression in both resistant Payette Russet and susceptible Russet Burbank at 24 hpi may indicate that terpene synthesis is a conserved early response to PVY infection. Together, this study and others have begun to characterize the spectrum of responses potato cultivars mount against specific PVY-strains, although additional studies are needed to better understand the molecular mechanisms that limit virus infections. In the long term, identification of additional virus limiting mechanisms and the genes involved may be useful to potato breeding programs.

#### 3.1.2. Upper, Uninoculated Russet Burbank Leaves Exhibit Different Transcriptional Responses before and after Systemic Infection

The potato cultivar Russet Burbank is consistently one of the most widely grown potato cultivars in North America due to its consistent tuber shape and cooking qualities [39]. Russet Burbank is also very susceptible to most strains of PVY. Characterization of the transcriptional responses to PVY in Russet Burbank will provide a better understanding of the effects of PVY infection on Russet Burbank, as well as identify genes that may facilitate PVY replication and spread. Thus, the transcriptional response to PVY was further assessed over a time course of infection, including the previously discussed 24 hpi time point and additional time points (i.e., 1 and 4 wpi). Systemic infection of PVY takes approximately four weeks to develop in Russet Burbank plants [40]. For these additional time points, PVY samples were obtained from leaves distal to the inoculation site since the effects of PVY infection on distal yet uninfected regions of potato plants is not well understood, as many previous sequencing efforts have focused on the early responses (i.e., 6 hpi to 5 days post-inoculation) of inoculated leaves [12,64].

At 1 wpi, transcriptional level changes were abundant, although PVY-infected Russet Burbank plants did not show visible symptoms of virus infection and virus levels in noninoculated leaves were below the detection limit of qPCR (i.e., <1000 RNA copies per 40 ng RNA) (Supplemental File S1B). Over 2500 genes were differentially expressed in the upper, noninoculated leaves of PVY-infected Russet Burbank plants compared to mock-infected plants. A similar number of genes exhibited either higher or lower expression (1359 up, 1307 down) (Figure 1B, Supplemental File S1J,K). Gene ontology analysis of the genes that exhibited lower expression indicated an enrichment in genes involved in RNA binding, methyltransferase activity, and the chloroplast (Figure 1B, Supplemental File S1L). GO analysis also indicated that DEGs with greater expression were involved in processes related to chloroplast and plastid organization and function. Plant viruses have a wide range of effects on photosynthesis and the photosynthetic machinery of plants [64,71,72]. Many plant viruses, including potyviruses such as PVY, use chloroplast membranes as replication structures [71]. Processes including protein modification and phosphorylation, ubiquitination, and plant–pathogen interactions were enriched among DEGs with decreased transcript abundance in PVY-infected plants at 1 wpi (Figure 1B). It may be that reduced expression of defense-related genes facilitates PVY spread.

Systemic infection of PVY in Russet Burbank results in the widespread accumulation of viral RNA genomes, transcripts, and proteins in leaf tissue throughout the plant. At 4 wpi, the PVY^N-Wi^-inoculated Russet Burbank plants were systemically infected and had a high viral burden (i.e., average of 7.5 × 10^6^ PVY-RNA copies per 40 ng RNA) (Supplemental File S1A). At 4 wpi, ~30% of the sequencing reads were viral in origin (Supplemental File S1B). Differential gene expression analysis indicated that slightly more differentially expressed genes exhibited decreased expression (2734) than increased expression (2584) in upper, noninoculated leaf samples from PVY-infected Russet Burbank (Figure 1B, Supplemental File S1M,N) (adj. *p*-value < 0.05). Gene ontology analysis indicated that processes including photosynthesis, oxidation reduction, and small molecule metabolism were enriched among genes that showed increased expression in PVY-infected plants, whereas biological processes including ribosomal structure, translation, plant–pathogen interactions, and cellular signaling were enriched among transcripts that decreased in expression (Supplemental File S1O). Virus infection affects nearly every aspect of the plant cell. Infection by plant viruses often results in chlorosis or mosaic patterning in infected leaves. These symptoms are indicative of damage to chloroplasts or the photosynthetic machinery. Importantly, Russet Burbank plants in this study were largely asymptomatic and did not display any chlorosis or strong mosaic symptoms. Since PVY reached high levels of systemic infection without obvious phenotypic effects on the plants, infections in this study could be categorized as tolerant [13,73,74,75]. This description is also congruent with the gene expression changes observed in this study (i.e., lower expression of defense genes and higher expression of genes involved in photosynthesis and chloroplast function).

### 3.2. Differences and Similarities in Differentially Expressed Genes between Potato Cultivars and Time Points Reveal Patterns of Infection and Possible Antiviral Genes

Differentially expressed genes between time points and cultivars were analyzed to determine which of the genes were shared between time points or cultivars (Figure 1B, Supplemental File 1P–W). Genes that are differentially expressed in two or more treatments or between time points may indicate that those genes could be involved in potato antiviral defense or in roles that benefit the virus. Gene ontology analysis of common differentially expressed genes was also conducted to ascertain patterns of types of genes or pathways that may be involved in multiple time points.

#### 3.2.1. DEGs Shared between PVY-Infected Payette Russet and Russet Burbank May Indicate Antiviral Roles

Genes that were differentially expressed in both PVY-inoculated Payette Russet and Russet Burbank may be involved in antiviral immunity. The resistance response in Payette Russet likely happens early after recognition of the virus, while Russet Burbank does not mount a successful resistance response but likely still mounts some defense responses as the infection progresses. Payette Russet samples from 24 hpi and Russet Burbank samples from 1 wpi shared 425 differentially expressed genes, of which 256 were shared exclusively between the two groups (Figure 1B, Supplemental File S1P). Among these shared genes, there was gene ontology enrichment for protein serine/threonine kinase activities, plant–pathogen interactions, and ubiquitin–protein transferase activity (Supplemental File S1Q). Enrichment within these treatment groups was exclusive to genes that exhibited decreased expression. For example, the salicylic acid concentration-controlling gene, *NPR1*-like, decreased in expression in Payette Russet at 24 hpi and in Russet Burbank at 1 wpi and 4 wpi (Figure 3, Supplemental Files S1 and S1L). Although genes involved in defense responses and plant–pathogen interactions were enriched for both Payette Russet at 24 hpi and Russet Burbank at 1 wpi, that these enrichments occurred only among genes exhibiting decreases in expression may indicate that the stress of PVY infection at inoculated leaves has strong effects on distal, not yet infected regions of susceptible Russet Burbank plants. Leaf tissue in which transcription of defense genes is decreased may be more prone to PVY infection as the virus spreads systemically.

Gene ontology analysis of differentially expressed genes that increased in expression exclusively in Russet Burbank at 1 wpi identified enrichment of chloroplast and plastid-related genes, as well as genes involved in RNA binding and amino acid synthesis (Figure 3, Supplemental File S1L). Many potyviruses, including PVY, use chloroplast membranes as replication complexes [76,77]. Increased transcription of genes involved in chloroplast/plastid organization could be due to an increased need for both chloroplast membranes as well as an increased demand to produce amino acids for chloroplast production and for future translation of viral proteins. Chloroplasts are devoid of RNAi activity and, therefore, serve as a prime location for viral replication and translation activity [71].

More than 500 genes were differentially expressed in both Payette Russet (24 hpi) and Russet Burbank plants at 4 wpi, of which 414 were exclusively shared between these two groups. The exclusively shared gene list was enriched for genes involved in molecular functions including ATP binding, kinase activity, sequence-specific DNA binding, and phosphotransferase activity. Biological processes associated with DNA replication and break repair and the mini-chromosome maintenance complex, which is also involved in DNA replication, were also enriched (Supplemental File S1S).

#### 3.2.2. DEGs Shared between Russet Burbank Time Points May Be Important to Successfully Establishing PVY Infection or Involved in Antiviral Defense

Viruses are adept at manipulating cellular conditions to promote their own replication and spread [71,78,79,80,81]. Genetic engineering to limit interactions between viral proteins and the host proteins required for viral replication can also be a sustainable form of antiviral resistance [82]. Genes that were differentially expressed in Russet Burbank at both 1 wpi and 4 wpi may be important for establishing PVY infection or antiviral defense and could serve as targets for future PVY-resistance editing experiments. A total of 953 differentially expressed genes were shared between Russet Burbank at the 1 wpi and 4 wpi time points (Supplemental File S1T). Among the shared genes that exhibited increased expression, there was strong enrichment for the cellular components, plastid and chloroplast, and secondary metabolite biosynthesis, as well as enrichment for the carotenoid biosynthesis and carbon metabolism pathways [83] (Supplemental File S1U). Enrichment among shared genes that exhibited decreased expression included the defense response and kinase activity. These results indicate that an overall decrease in transcription of defense response genes and genes involved in kinase activity/phosphorylation cascades may be an important aspect of establishing PVY infection in Russet Burbank.

Beyond gene ontology, patterns of expression among individual genes or gene families among and between treatment groups provides additional information regarding the PVY infection process in Russet Burbank potato plants. Four genes encoding Heat shock protein 70 interacting proteins differentially exhibited increased in expression in PVY-infected Russet Burbank plants at both 1 wpi and 4 wpi (Supplemental File S1T). Heat shock protein 70 (HSP70) serves important pro-viral roles during plant virus infections. For example, successful infection by Tomato yellow leaf curl virus depends on interactions between tomato HSP70 and the viral coat protein [84]. The assembly of the replicase complex of Red clover necrotic mosaic virus also depends on interactions with HSP70 and HSP90 [85]. Interestingly, HSP70 is also required for successful deployment of plant defense responses in *Nicotiana benthamiana* [86]. The specific roles of HSP70 proteins are not well understood but they could play pivotal pro- or antiviral roles during PVY infection. Five genes encoding Kunitz family trypsin and protease inhibitor proteins also exhibited greater expression in PVY-infected Russet Burbank at 1 wpi and 4 wpi (Supplemental File S1T). Kunitz family trypsin and protease inhibitor proteins negatively regulate programmed cell death and the hypersensitive response in Arabidopsis in response to fungal and bacterial pathogens [87]. As the Russet Burbank plants remain mostly asymptomatic even when systemically infected with PVY, it is possible that these Kunitz family trypsin and protease inhibitor also negatively regulate defense responses to viral pathogens, though more research is needed to test this hypothesis.

### 3.3. Expression and Type of Small RNA Varies by Time Point and Cultivar

Small RNAs are employed by plants in myriad ways and influence nearly every aspect of plant biology [88]. The length of the small RNA in plants can, in part, dictate function. Non-coding RNAs with secondary stem loop structure can be processed by the RNAi machinery into microRNAs (miRNAs), which are often 21- or 22-nt in length. Argonaute-bound miRNAs bind complementary mRNA transcripts and regulate gene expression by either transcript cleavage or translational inhibition of the target mRNA [89]. MicroRNAs and other small RNAs functioning within the RNAi pathway in plants are generally involved in negative regulation of gene expression, particularly transcriptional and post-transcriptional gene silencing. Transcriptional regulation by DNA methylation is in part controlled by the targeting of complementary long, non-coding RNAs by Argonaute proteins bound to 24-nucleotide small RNAs, which are often the most common length of small RNA found in flowering plants [90,91].

**Figure 4:**
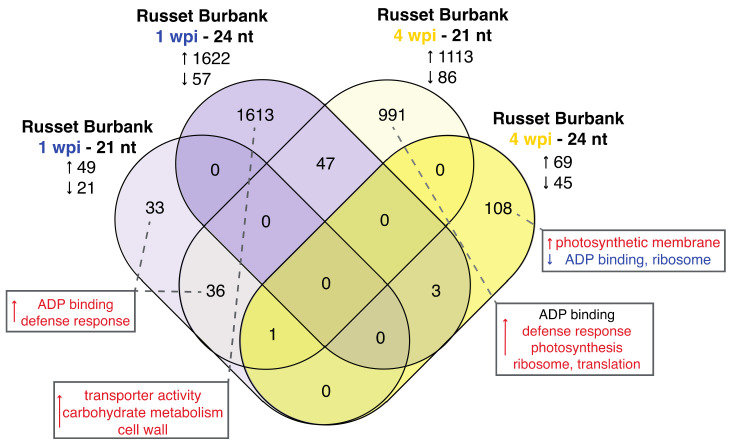
Potato small RNA transcriptional response to PVY-infection is time- and length-dependent. A Venn diagram depicts unique and shared differentially expressed 21- and 24-nucleotide small RNAs in Russet Burbank at 1 wpi (blue) and at 4 wpi (yellow) in response to PVY infection. No differentially expressed small RNAs were shared between all of the time points and cultivars. Over half of the 21-nucleotide small RNAs that increased in abundance at 1 wpi were also differentially expressed at 4 wpi. Most of the differentially expressed small RNAs at 1 wpi were 24 nucleotides long, while most of those differentially expressed at 4 wpi were 21 nucleotides long, with many fewer 24-nucelotide small RNAs at that time point. Gene ontology results indicate that defense response and ADP-binding were enriched among all differentially expressed 21-nucleotide small RNAs, while enrichment for photosynthesis, ribosome- and translation-associated genes were enriched in the 21-nucleotide small RNAs at 4 wpi only. Transporter activity, carbohydrate metabolism, and the cell wall were enriched among 24-nucleotide small RNAs at 1 wpi, with the photosynthetic membrane, ADP-binding, and the ribosomes were enriched among 24-nucleotide small RNAs at 4 wpi. Full lists of differentially expressed genes and their corresponding fold changes are reported in Supplemental File S2.

While ~40% of the small RNAs in systemically PVY-infected Russet Burbank plants at 4 wpi aligned to the PVY genome, the focus of this analysis was primarily on 21–24 nucleotides (nt) small RNAs that aligned within gene regions of the potato genome (Supplemental File S2A), although 24-nt small RNAs that align outside of gene regions can also impact transcription by binding to promoter regions or repress transcription of transposable elements [51]. We identified abundant clusters of 21–24-nt small RNAs with Shortstack that were likely biologically relevant through the RNAi pathway [51]. The majority of the differentially abundant small RNAs that aligned to genes in Russet Burbank were either 21- or 24-nt small RNAs, which is typical of flowering plants (Figure 4, Supplemental File S2B,D) [51]. Interestingly, the majority of differentially expressed small RNAs were 24-nt in length at 1 wpi and 21-nt in length at 4 wpi, and the abundance of almost all these small RNAs increased (Supplemental File S2B,D). At 1 wpi, the genes aligning to the 24-nt small RNAs, that were less abundant in virus-infected plants compared to mock-infected controls, were enriched for transporter activity, carbohydrate metabolism, and cell wall/cell periphery (Supplemental File S2C). Many of the 21-nt small RNAs that varied in abundance at 1 wpi aligned to NLR resistance genes, which are typically subject to negative regulation by small RNAs. Further, approximately half of those genes also aligned with 21-nt small RNAs that were abundant at 4 wpi (Figure 4). At 1 wpi, the genes that aligned to the differentially expressed 21-nt RNAs were enrichment for ADP-binding and defense responses.

The small RNAs in Russet Burbank plants with systemic PVY infections (4 wpi) aligned to numerous host genes (Supplemental File S2D). Specifically, 21-nucleotide small RNAs aligned with over 1000 genes and the 24-nt RNAs aligned to over 100 genes, and GO analysis was carried out on the genes that aligned with these small RNAs. The genes that aligned to 21-nt small RNAs that exhibited both increased and decreased abundance were enriched for genes with ADP-binding activity, whereas the genes that aligned to 21-nt RNAs with greater abundance were enriched for genes involved in defense responses, photosynthesis, ribosomes, and translation (Supplemental File S2E). Similar enrichment of host genes that may be targeted by PVY-derived small RNAs were identified in Russet Burbank at 4 wpi, as part of a study solely focused on virus-derived small RNAs [92]. Enrichment analysis of the genes that aligned to the 24-nt small RNA clusters that were more abundant in PVY-infected plants revealed an enrichment in photosynthetic membrane associated genes. Conversely, ADP-binding and ribosome associated genes were enriched among those clusters that displayed decreases in expression.

In some instances, the observed increased abundance in small RNAs that aligned to potato genes inversely corresponded to changes in mRNA abundance. For example, Russet Burbank genes that exhibited decreased expression at 1 wpi and 4 wpi included a higher number of genes involved in defense responses than would be expected by chance (Supplemental File S1C,E). Correspondingly, the abundance of small RNAs targeting defense genes was greater at 1 wpi and 4 wpi. Intuitively, these results make sense, as increases in small RNAs would, in theory, result in decreases in target gene transcript. However, this pattern was not observed in all the results, as enrichment for ribosomal processes/translation existed among both mRNA and small RNAs that decreased in expression/abundance.

The roles of small RNAs in regulating defense responses and other physiological traits are well established, but the regulation of ribosomal proteins is much less well understood. Plant small RNAs target transcripts from NLR genes and tightly regulate the expression of plant immune responses [24,93,94]. We analyzed the composition of genes that aligned with small RNAs in PVY-infected Russet Burbank plants. At 1 wpi, 35.2% (25/71) of the differentially abundant 21-nt small RNAs aligned with NLR-type disease resistance genes (Supplemental File S2B). Similarly, of the differentially abundant 21-nt small RNAs at 4 wpi, 21.8% (240/1099) aligned to annotated disease resistance genes (Supplemental File S2D). At 4 wpi, all of the 21-nt small RNA clusters that were more abundant in PVY-infected plants aligned to disease resistance genes. The potato genome encodes 786 NLR-type disease resistance genes, therefore, ~32% of the total disease resistance genes were increasingly targeted by 21-nt small RNAs during systemic PVY infection at 4 wpi [53,95]. This indicates that small RNAs and RNAi-mediated gene regulation may be important for limiting the antiviral responses mounted by Russet Burbank potato plants against PVY.

There were distinct differences in the small RNA composition in Russet Burbank plants at 1 week post PVY-infection compared to 4 wpi, when the plants were systemically infected. These differences were striking, as the 1 wpi time point was dominated by differences in 24-nt small RNA abundance and 4 wpi was dominated by differences in the 21-nt small RNAs (Figure 4). DNA-methylation, as conferred in part by the 24-nt small RNA pathway, often targets transposable elements to prevent expression [96]. It is possible that PVY infection also induces transposable element release and that this causes the rise increase in 24-nt small RNAs observed in this study.

#### 3.3.1. The miRNA Profiles of PVY-Infected Russet Burbank Plants Mirrors mRNA Results and Is Similar to PVY-Tolerant Potato Cultivars

MicroRNAs are not well-annotated within the potato genome and many miRNA identification software programs are prone to high rates of false positive annotations [97]. Shortstack is a conservative predictor of putative miRNAs. Because of the conservative nature of Shortstack and the relative lack of miRNA annotation in the potato genome, we decided to analyze the abundance of small RNAs that were both identified as miRNAs by Shortstack and similar in sequence to annotated plant miRNAs.

A total of 20 miRNAs that met our requirements were identified in the sequencing libraries generated from Russet Burbank plants at 1 wpi (Supplemental File S2H). Of those 20 miRNAs, 19 were more abundant in PVY-infected plants compared to non-infected plants. Two miRNAs that were much more abundant in PVY-infected plants most closely resembled miR7992 and miR6149, both of which exhibited complementarity to regions of the PVY RNA genome and could in theory target PVY [98]. MiR162 was one of the most abundant miRNAs in PVY-infected plants and regulates the expression of *DICER1-LIKE*, which is a key component of the plant miRNA pathway in Arabidopsis [99]. Mir482, which targets NLR transcripts, was the most abundant miRNA at 1 wpi. At 4 wpi, we identified 25 miRNAs that fit our criteria (Supplemental File S2I). Of those miRNAs, 24 were more abundant in PVY-infected plants compared to non-infected Russet Burbank plants. The most abundant miRNA belonged to a mir167 miRNA family, which target transcripts involved in plant growth regulation [100]. Multiple miRNAs annotated as species of miR408 were more abundant in PVY-infected plants at 4 wpi. The greater abundance of miR408 may result in attenuated immune responses, as similar results have been described in sweet potato [100,101].

Understanding tolerance to virus infection has recently received increased scientific attention, as tolerant plants are less likely to put strong evolutionary pressures on viruses to evolve mechanisms or structures that render resistance genes and immune responses ineffective [75,102]. However, tolerant plants can harbor very high viral loads and, thus, can serve as viral reservoirs in fields, potentially seeding outbreaks to neighboring, more susceptible plants and allowing for mixed infections and possible recombination events between different viral strains [13]. Recent research indicated that up-regulation of key miRNAs in a tolerant potato cultivar, Desiree, may assist in establishing a tolerant infection to PVY^NTN^ [74]. Our results corroborate some of these findings, as miR167, miR391, miR171, miR164, and miR390 were all differentially increased in expression at either 1 wpi or 4 wpi in PVY^N-Wi^-infected Russet Burbank plants (Supplemental File S2H,I). These miRNAs have been implicated in facilitating tolerance and mutualism in host plants. That we found similar results in a tolerant infection with a different potato cultivar (i.e., Russet Burbank) and different PVY strain (i.e., PVY^N-Wi^), further strengthens previous findings [74]. Symptoms of PVY infection can vary with environmental conditions and while we observed tolerant-like infections in Russet Burbank plants grown from sterile tissue culture in growth chambers, symptomatic infections can develop in field conditions [40].

#### 3.3.2. Analysis of phasiRNAs in PVY-Infected Potato Plants Reveals Enrichment of NLR Resistance Gene Targets and Similarities between Time Points

**Figure 5:**
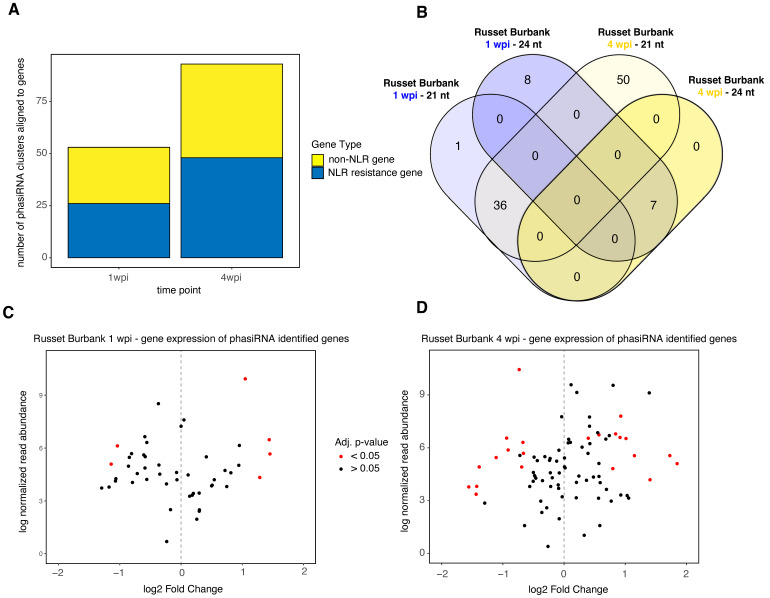
Phased short-interfering RNAs predominantly target NLR proteins in Russet Burbank and are not correlated with expression patterns of mRNAs. (**A**) PhasiRNAs separated by time point and gene type. Approximately half (25/52 at 1 wpi, 48/93 at 4wpi) of the phasiRNAs aligned to annotated NLR resistance genes at each time point (blue); whereas clusters that did not align to NLR resistance genes are in yellow. (**B**) Similar phasiRNAs are shared between time points and among small RNA lengths. All but one of the 21-nucleotide phasiRNAs identified at 1 wpi was also identified at 4 wpi (blue). Similarly, all seven 24-nucleotide phasiRNAs at 4 wpi were also identified as phasiRNAs at 1 wpi (yellow). (**C**,**D**) Normalized expression of genes targeted by phasiRNAs colored by state of differential expression. Genes targeted by phasiRNAs do not exhibit a correlation with either increased or decreased expression. Full lists of differentially expressed phasiRNAs and their corresponding fold changes are reported in Supplemental File S2.

In plants, AGO proteins loaded with distinct 22-nt small RNAs can trigger phased cleavage of a target RNA by dicer-like proteins, ultimately resulting in the production of many small RNAs (phased siRNAs, or phasiRNAs) from a single transcript. The resulting phasiRNAs can be employed to target other transcripts produced from the same gene or locus, or other transcripts with similar target regions [55]. PhasiRNA production results in distinctive alignment signatures that can be computationally identified by programs such as Shortstack [49,97,103]. We identified putative phasiRNAs within the small RNA alignments of Russet Burbank at 1 wpi and 4 wpi using Shortstack to gain a better understanding of how phasiRNA are regulated in response to PVY infection in susceptible plants. Analysis of phasiRNAs in PVY-infected Russet Burbank plants at 1 wpi resulted in 52 phasiRNA loci from gene regions, with 37 (71%) of those loci composed of 21-nt phasiRNAs and the remaining 15 (29%) phasiRNA loci composed of 24-nt phasiRNAs (Figure 5A,B, Supplemental File S2J). Shortstack analysis of the PVY-infected Russet Burbank plants at 4 wpi resulted in 104 phasiRNA loci, which were composed of a mixture of 21-nucleotide (92%) and 24-nucleotide (8%) small RNA clusters (Supplemental File S2K).

PhasiRNA production aids in the regulation of defense-related genes [55]. The 21-nt phasiRNAs aligned to predominantly NLR genes and other defense-related genes at both time points in PVY-infected plants, while the 24-nt phasiRNAs did not align to any NLR genes but did align to transcripts from a protein kinase superfamily protein (Soltu.DM.07G005020) and a Kunitz family trypsin and protease inhibitor protein (Soltu.DM.03G023530). Nearly half (26/52) of the phasiRNA loci at 1 wpi aligned to NLR-type resistance genes (Figure 5B). Nearly twice as many phasiRNA alignment loci were detected at 4 wpi (93) in Russet Burbank than at 1 wpi. The types of genes that phasiRNAs at 4 wpi aligned to were similar in type and proportion as at 1 wpi, as slightly more than half (48/93) aligned to NLR-type resistance genes. The non-NLR resistance genes that contained phasiRNA alignments consisted of other defense-related genes, including *Dicer2-like* (Soltu.DM.11G004150) and a Kunitz family trypsin and protease inhibitor (Soltu.DM.03G023530), both of which are involved in antiviral defense or defense responses in plants against other pathogens [104]. These results indicate that while NLR-type resistance genes are the primary target of phasiRNAs, they are not the only type of defense-related genes that are targeted by phasiRNA in potato in response to PVY infection.

We earlier described that the transcriptional responses in Payette Russet and Russet Burbank exhibited some similarities between time points, but that the majority of the differentially expressed genes were distinct to that cultivar and time point. We hypothesized that phasiRNA targets may also segregate by time point in Russet Burbank at 1 wpi and 4 wpi. All but one of the genes aligned with 21-nt phasiRNAs was also targeted byphasiRNAs at 4 wpi, while 12 phasiRNA loci were unique to 4 wpi. All seven of the 24-nt phasiRNA loci detected at 4 wpi were also targeted by 24-nt phasiRNAs at 1 wpi, while the remaining eight 24-nt phasiRNA loci were unique to 1 wpi (Figure 5B). These results indicate that phasiRNAs do not necessarily match the transcriptional patterns as observed in the mRNA data, but that phasiRNA may target similar genes at different time points during PVY infection of Russet Burbank.

Post-transcriptional regulation by phasiRNAs can result in decreases in target transcripts [55]. In this study, detected phasiRNA presence did not exhibit strong correlation with changes in abundance among affiliated genes (Figure 5C,D). Many of the genes which had phasiRNA alignments were not differentially expressed or exhibited differential expression in either a positive or negative direction at both 1 wpi and 4 wpi (Figure 5C,D). These results indicate that detection of phasiRNAs via sequencing alignments does not necessarily correlate with reduced abundance of target mRNAs at a given time point post-infection; however, additional time points may be required to capture the dynamic relationship between phasiRNAs and transcript abundance.

The phasiRNAs identified in this study, and their corresponding genes, may be involved in either promoting or reducing PVY infections in Russet Burbank plants. In this study, we identified a poly(A) binding protein (Soltu.DM.01G000470) transcript that was less abundant in PVY-infected plants, compared to uninfected controls, that was the target of a phasiRNA cluster that was more abundant in PVY-infected plants. Poly (A) binding proteins are necessary for replication of other potyviruses, but their involvement in in PVY replication has not yet been described [105]. Our transcriptome and small RNA analysis suggest that poly (A) binding protein may be important for PVY infection. Genes that produce proteins required for PVY replication and spread could be targets for gene editing techniques to alter viral protein-host–protein interaction sites and, thus, may be useful in the development of PVY-resistant plants [82].

### 3.4. Alternative Splicing Analysis Reveals Genes outside of Differential Expression Analysis with Potential Impacts on PVY Infection

Most transcripts in eukaryotes can be alternatively spliced [106,107]. Alternative splicing allows for a single transcript to produce a diversity of protein structures and functions while also providing another avenue to regulate gene expression [65,106,108]. Patterns of alternative splicing can be influenced both by tissue and cell type but can also be a component of a response to environmental stimuli, including regulation of defense responses to invading pathogens. Alternative splicing is a relatively understudied area of host responses to plant viruses. To date there have been no studies published detailing the alternative splicing landscape in host plants following PVY infection. Therefore, we used the R package, IsoformSwitchAnalyzeR, to identify and analyze alternative splicing patterns following PVY infection in both Payette Russet and Russet Burbank potato cultivars [109]. Alternative splicing analysis of the transcriptomic libraries indicated that hundreds of genes had transcripts that were alternatively spliced in response to PVY infection in both cultivars (adj. *p*-value < 0.05) (Supplemental File S3A–D).

**Figure 6:**
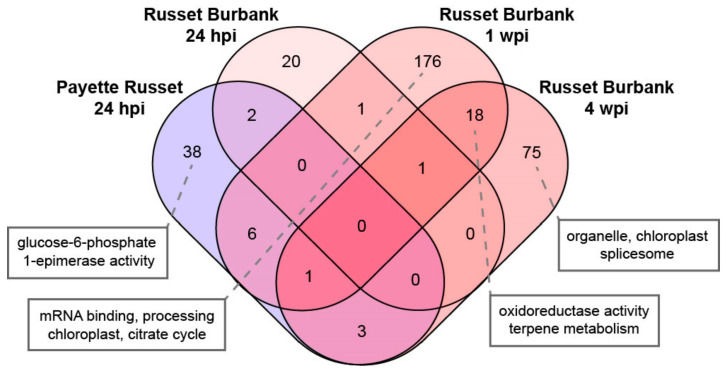
Alternative splicing analysis in Payette Russet and Russet Burbank potato cultivars in response to PVY infection. Hundreds of genes differentially spliced genes were identified in this study, some of which shared splicing patterns between time points. Gene ontology analysis of the spliced genes revealed glucose-6-phosphate enrichment among differentially spliced genes in Payette Russet at 24 hpi. The highest number of differential splicing occurred at 1 wpi in Russet Burbank, with over 200 genes that were likely differentially spliced and ontology enrichment for mRNA binding/processing, chloroplast, and the citrate cycle.

Within Payette Russet samples at 24 hpi, 49 genes exhibited differential splicing patterns in PVY-inoculated leaves as compared to mock-inoculated plants (Figure 6, Supplemental File S3A). The transcripts that exhibited the greatest statistical strength of differential splicing belonged to an ARM repeat superfamily protein (Soltu.DM.05G013700), a potassium (K+) uptake permease (Soltu.DM.02G003540), a protein of unknown function containing a DUF538 domain (Soltu.DM.12G024860), and a glycine-rich RNA-binding protein (Soltu.DM.02G030950). Gene ontology analysis of the differentially spliced genes determined that there was enrichment for genes involved in glucose-6-phosphate 1-epimerase activity (Supplemental File S3E). PVY-infected Russet Burbank leaves exhibited 24, 203, and 81 alternatively spliced genes at 24 hpi and 1 and 4 wpi as compared to mock-inoculated plants (Figure 6, Supplemental File S3B–D). Gene ontology analysis of differentially spliced genes at 1 wpi revealed enrichment of genes involved in mRNA-binding/processing, chloroplast function, and the citrate cycle (Supplemental File S3F). Differentially spliced genes associated with chloroplasts, organelles, and the splicesome were enriched at 4 wpi (Supplemental File S3G). A total of 18 genes were differentially spliced in PVY-infected Russet Burbank at both 1 wpi and 4 wpi and gene ontology enrichment revealed enrichment for oxidoreductase and terpene metabolism (Supplemental File S3H)

**Figure 7:**
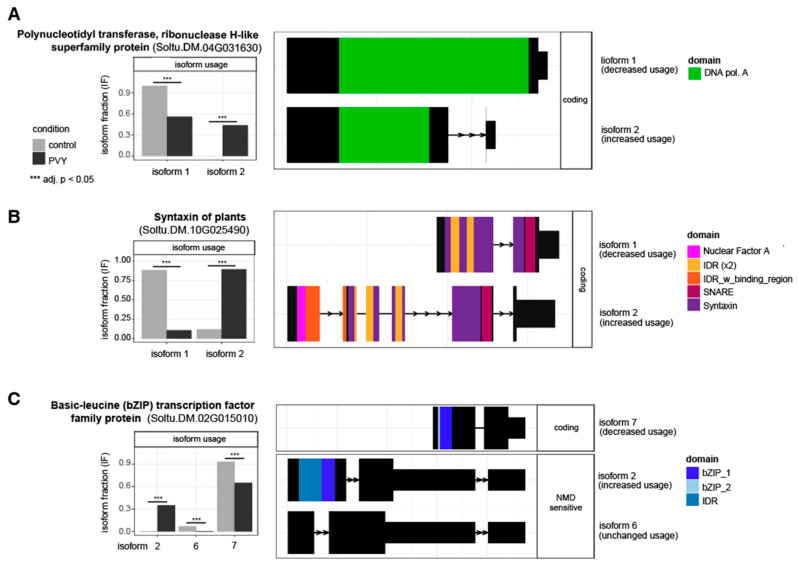
Alternative splicing profiles of select genes in PVY-infected Russet Burbank plants. (**A**) Isoform 2 of the gene *Polynucleotidyl transferase, ribonuclease H-like superfamily protein* (Soltu.DM.04G031630) exhibited increased usage in PVY-infected Russet Burbank plants at 1 wpi, resulting in a higher proportion of transcripts encoding a truncated C-terminal end, which may be important for an effective RNA interference response. (**B**) Isoform 2 of the gene *Syntaxin of plants* (Soltu.DM.10G025490) exhibits increased usage relative to isoform 1 in PVY-infected Russet Burbank plants at 4 wpi. (**C**) Isoform 2 of the gene *Basic-leucine (bZIP) transcription factor family protein* (Soltu.DM.02G015010), which contains an intrinsically disordered domain, exhibits increased usage at 4 wpi in PVY-infected Russet Burbank plants.

At 1 wpi, the transcripts with the most statistical strength for differential splicing belonged to genes encoding a Polynucleotidyl transferase, ribonuclease H-like superfamily protein (Soltu.DM.04G031630), and a basic leucine (bZIP) transcription factor family protein (Soltu.DM.08G019590) (Supplemental File S3C). The Polynucleotidyl transferase, ribonuclease H-like superfamily protein may also be annotated as a Werner Syndrome-like exonuclease (WEX). Some WEX proteins act as cofactors to Argonaute proteins in Arabidopsis and are critical to RISC function. These proteins degrade uridylated 5′ single-stranded RNAs cleaved by Argonaute proteins, thus, allowing for RISC to target other transcripts [110]. Isoform 2 of the ribonuclease H-like superfamily protein is truncated at the C-terminal end and would theoretically be missing components of catalytic domains necessary for proper functioning of RISC (Figure 7A). RNA interference is a primary antiviral defense pathway in plants; therefore, dysfunctional RISCs would render a plant more susceptible to viral infection. That the alternative splicing of this transcript occurs at 1 wpi in leaves that are yet to be infected suggests that PVY may be able to alter the state of uninfected cells to facilitate incoming infection. Plants also rely extensively on the intercellular trafficking of virus-derived small RNAs from infected tissue to distal cells to ‘prime’ those cells for impending virus infection. Interference with RISC function in distal, yet to be infected tissues could prevent priming of uninfected cells. The alternative splicing of this *Polynucleotidyl transferase, ribonuclease H-like superfamily* gene will require further investigation to understand its impact during PVY infection.

One gene which exhibited differential splicing at 4 wpi in Russet Burbank was *syntaxin of plants* (Soltu.DM.10G025490), homologous to the Arabidopsis *syntaxin of plants 121* (SYP121). SYP121 proteins are critical components of SNARE complexes, which control bulk transport and vesicle traffic in the cell (Figure 7B) [111]. Syntaxin proteins are also involved in various defense pathways in Arabidopsis [112]. Isoform 1 was less abundant in PVY-infected plants, while isoform 2 levels were higher. Isoform 2 differs from isoform 1 in that it contains an N-terminal Nuclear factor YA domain and an intrinsically disordered domain with a binding region. Nuclear factor Y binding domains in plants are generally individually encoded in different gene families and oligomerize in response to various stressors to form a transcription factor complex recognizing the CCAAT box in target gene promoters. It is possible that differential splicing of *SYNTAXIN OF PLANTS* in potato to include the Nuclear factor YA domain results in virus-specific regulation of defense responses, but further research is necessary to gain a better understanding.

Consistent patterns of alternative splicing across time points in PVY-infected plants may be indicative of specific splicing patterns that are important for PVY infection. The basic leucine zipper (bZIP) transcription factor family protein (Soltu.DM.08G019590) exhibits sequence similarity to Arabidopsis bZIP17, which regulates salt stress responses in Arabidopsis [113]. The transcript Isoform 2 was more abundant in PVY-infected Russet Burbank plants at 1 wpi and 4 wpi, while isoform 7 was less prevalent at both time points (Figure 7C). The two transcripts differ in that isoform 2 has an N-terminal intrinsically disordered domain and is missing a second basic leucine zipper domain that is present in isoform 7. The functional ramifications of this alternative splicing pattern are not understood, but often intrinsically disordered regions within transcription factors are important to facilitating interactions with DNA and gene regulation [114,115,116]. It is possible that the potato bZIP protein regulates immune genes in response to PVY infection, but further studies are needed to investigate possible defense roles or pro-viral roles. Transcripts from other potentially interesting genes were differentially spliced at both 1 wpi and 4 wpi in Russet Burbank (silencing defective, Soltu.DM.07G024420; RNA-binding (RRM/RBD/RNP motifs family protein, Soltu.DM.01G005860) and may be promising targets of future research on potato–PVY interactions as well.

**Figure 8:**
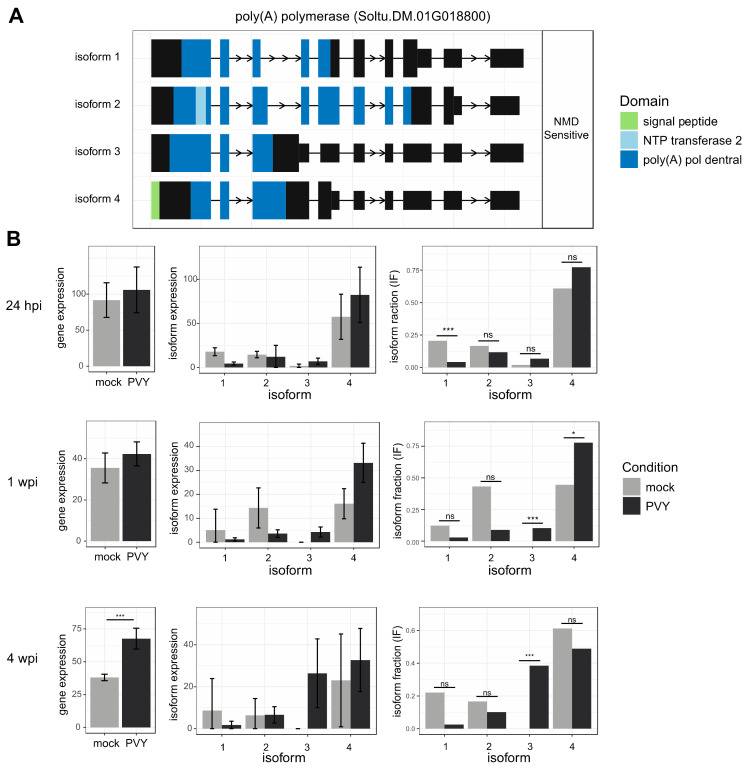
Poly(A) polymerase exhibits alternative splicing at 24 hpi, 1 wpi, and 4 wpi in PVY-infected Russet Burbank plants. Transcript isoforms of the potato gene, poly(A) polymerase (Soltu.DM.01G018800) exhibit similar differential splicing patterns in all sampled time points of Russet Burbank during PVY infection. (**A**) A splicing graph depicting the four different isoforms of poly(A) polymerase expressed in Russet Burbank. Isoform 2 contains an NTP transferase 2 domain and isoform 4 contains a signal peptide domain. (**B**) Gene expression, isoform expression, and isoform usage graphs for poly(A) polymerase at 24 hpi, 1 wpi, and 4 wpi in Russet Burbank plants. All four isoforms were expressed at 24 hpi, 1 wpi, and 4 wpi. Isoform 2 contains an NTP transferase 2 domain. Poly(A) polymerase is differentially expressed at 4 wpi but not at 24 hpi or 1 wpi. This increase in expression is likely due to an increase in expression of isoform 3, which also exhibits a significant increase in isoform usage at 4 wpi. In this figure * *p* < 0.05; *** *p* < 0.0005; and “ns” indicates non-significant.

The only gene that was differentially spliced in Russet Burbank at all three time points was poly(A) polymerase (Soltu.DM.01G018800) (Figure 8). Poly(A) polymerases enhance transcript stability and translational efficiency by adenylating the 3′ ends of pre-mRNAs [117]. The potato genome encodes multiple poly(A) polymerases and one of them (i.e., Soltu.DM.01G018800) has four different isoforms. All four isoforms were expressed at 24 hpi, 1 wpi, and 4 wpi (Figure 8). Isoform 2 contains an NTP transferase 2 domain, which is not found in the other three isoforms, while isoform 4 is the only isoform to contain a signal peptide. Poly(A) polymerase was differentially expressed at 4 wpi, but not at 24 hpi or 1 wpi. The increase in expression was likely due to increased expression of isoform 3. All four isoforms are sensitive to nonsense mediated decay, indicating that it is possible that some or all of the transcripts described may not be translated into functional proteins, as the NMD pathway does not automatically degrade all transcripts with pre-mature stop codons [118]. The analysis of alternatively spliced transcripts adds another dimension of information to our growing understanding of the interactions between potato cultivars and PVY. Although additional studies are required, the genes and isoforms identified in this study may play key roles in host responses to PVY infection.

## 4. Conclusions

To gain a better understanding of potato antiviral responses, we examined the transcript and small RNA profiles of PVY^N-Wi^-infected Russet Burbank plants at three time points post-infection which included the development of systemic infection, and in one matching early time point post-inoculation (i.e., 24 hpi) in Payette Russet, a cultivar that exhibits extreme PVY-resistance. We identified thousands of differentially expressed genes which varied by cultivar and time point. Increased expression of terpene synthase, heat shock protein, and kinase encoding genes in the context of PVY-infection in both cultivars may indicate antiviral roles of these genes, though additional studies are needed. The majority of DEGs in PVY^N-Wi^-inoculated Payette Russet plants were down-regulated at 24 hpi. Gene ontology analysis indicated that defense responses were enriched among genes with lower expression levels, while metabolic processes were enriched among genes with greater expression in PVY^N-Wi^-infected plants. While a lower total number of DEGs were identified in PVY-susceptible Russet Burbank plants at 24 hpi, the majority (i.e., 64/100) of them exhibited lower expression in virus-infected plants. At later time points (1 wpi, 4 wpi) post PVY^N-Wi^-infection of Russet Burbank plants thousands of DEGs were identified, with an approximately equal number of up- and down-regulated genes. Genes with increased expression included those involved in photosynthesis, chloroplast function, and secondary metabolite production, and the heat shock response. In addition, genes encoding Kunitz family trypsin and protease inhibitor proteins exhibited increased expression and may play a role in limiting HR in potato and, in turn, facilitating virus spread throughout the plant. Differential gene expression varied by cultivar and time point, and also varies with PVY-strain and environmental conditions, therefore, this work together with previous and future studies on gene regulation in potato plants will enhance our understanding of effective anti-PVY response mechanisms. We identified differential expression of genes in distal yet uninfected regions of the plant, indicating PVY-infection has a considerable influence on overall gene expression. Additional studies of systemic PVY-infection in susceptible cultivars will enhance our understanding of the development of antiviral responses throughout the infection process. As part of our bioinformatic analysis, transcript sequences from the most recent annotation of the potato genome (i.e., DM version 6.1) were utilized in BLAST searches against potato, wild potato species commonly used for breeding purposes, and other organisms. We reported the top hits of these searches in Supplemental Files S1 and S4 to provide a link between the potato genome online resources and NCBI resources to facilitate comparative analyses between the potato genes/proteins with other organisms. Analyses of small RNAs including 21- and 24-nt small RNAs and miRNAs from the same time points indicate similarities to tolerant infections in other potato cultivars, expanding upon and further solidifying the results of the few other studies that have examined small RNAs in PVY-infected potato [73,74,102]. Tolerant plants may serve as viral reservoirs that may produce recombinant viruses and/or outbreaks and, therefore, tolerance to PVY infection is not a useful trait for potato breeders. However, understanding the mechanisms of tolerance could aid in the engineering of beneficial or mutualistic relationships to improve crop yield and/or decrease nutrient input uses in potato and other important crop species [119,120].

This study is the first to examine alternative splicing, an important regulator of biological processes, in PVY-infected potato plants. Our data indicate that differential splicing is prevalent, and analyses of alternatively spliced genes included a BZIP transcription factor which may promote the expression of genes involved in antiviral defense. This study also includes the first analysis of phasiRNAs in PVY-infected Russet Burbank plants, we determined that more than half of the phasiRNAs aligned to NLR (nucleotide binding, leucine-rich repeat proteins indicating that NLR expression is post-transcriptionally down-regulated by the miRNA pathway, and that down-regulation is amplified by phasiRNA production. PhasiRNA results were similar at 1 and 4 wpi in susceptible Russet Burbank plants indicating that this response is systemic and occurs even at times of low to undetectable levels of PVY infection in uninoculated leaves (i.e., at 1 wpi), and during systemic infection with high viral loads. Although phasiRNA abundance did not always correlate with mRNA expression data at particular time points, subtle differences in NLR expression may be biologically significant. Future studies with additional time points are needed to examine the dynamic nature of phasiRNAs and their target mRNAs more fully. These studies, as well as those that examine transcriptional responses to PVY, including alternative splicing, will provide greater understanding of potato virus limiting defense mechanisms that may benefit breeding programs in the future.

## Data Availability

All data is available as associated Supplemental Materials and sequence data was deposited NCBI Sequence Read Archive under submission number PRJNA768797. Additional information is available upon request.

## Supplementary Materials

The following supporting information can be downloaded at: https://www.mdpi.com/article/10.3390/v14030523/s1, Supplemental Figure S1. qPCR validation of select genes in Payette Russet and Russet Burbank plants at 24 hpi; Supplemental Figure S2. qPCR validation of select genes in Russet Burbank plants at 1 and 4 wpi; Supplemental Figure S3. PVY^N-Wi^ sequences from this study shares 99.7% nucleotide identity with PVY^N-Wi^ reference genome (HQ912863); and Supplemental Files S1–S4 include all the analyzed sequencing results.
