# Supplementary material for: Transcriptome and Small RNA Profiling of Potato Virus Y Infected Potato Cultivars, Including Systemically Infected Russet Burbank
Source: Viruses. 2022 Mar 3;14(3):523. doi: 10.3390/v14030523 (PMC8952017; doi:10.3390/v14030523)
Supplement: Supplementary file 1 [file viruses-14-00523-s001.zip › viruses-1593488-suppl/viruses-1593488-supplementary.pdf]

Transcriptome and Small RNA Profiling of Potato virus Y Infected Potato Cultivars, including Systemically Infected Russet Burbank

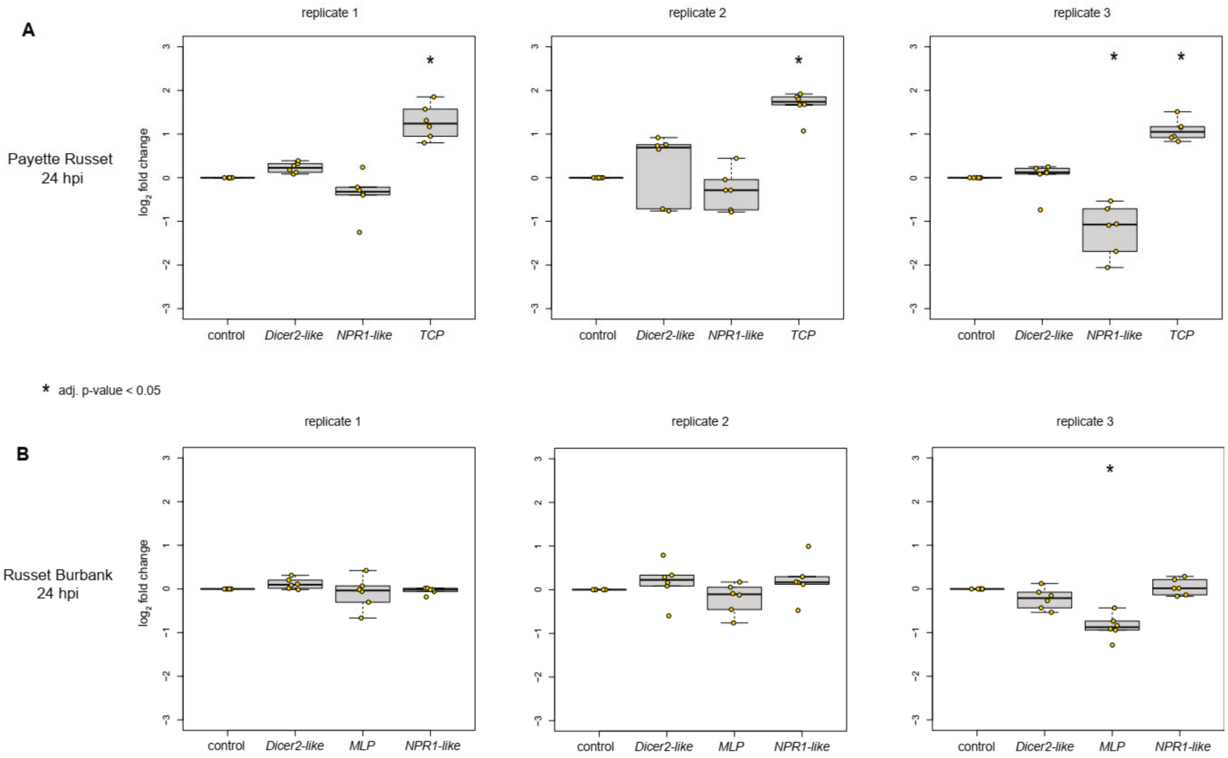

Supplemental Figure S1. qPCR validation of select genes in Payette Russet and Russet Burbank plants at 24 hpi. (**A**) qPCR gene expression results from three biological replicates in PVY-inoculated Payette Russet plants support transcriptome data. Specifically, *Dicer2-like* (Soltu.DM.11G004150) expression levels were similar to levels in mock-infected control plants (0.22, 0.42, 0.04 log_2 fold change); *NPR1-like* (Soltu.DM.07G014680) expression was decreased in biological replicate 3 (-1.11 log_2 fold change) and, although not supported by statistical analysis, trended lower in biological replicates 1 (-0.31 log_2 fold change) and 2 (-0.22 log_2 fold change); and *TCP* (Soltu.DM.04G011720) exhibited increased in expression in all three biological replicates(1.32, 1.69, 1.11 log_2 fold change). (**B**) PVY-inoculated Russet Burbank plant exhibited lower *MLP* (Soltu.DM.04G002870) expression in biological replicate 3 (-0.84 log_2 fold change), while no changes in gene expression were observed for *MLP* (-0.06, -0.17 log_2 fold change) in the other two replicates, *NPR1-like* (-0.03, 0.27, 0.05 log_2 fold change), or *Dicer2-like* (0.12, 0.23, -0.21 log_2 fold change) in any of the replicates. The RNA sequencing results suggested a decrease in expression of *MLP* (-1.58 log_2 fold change), but no changes for either *NPR1-like* or *Dicer2-like*. Statistical differences in gene expression between mock-infected and PVY-infected potato plants (n = 6 per treatment group per biological replicate) were performed using Wilcoxon ranked sum test, *p<0.05.

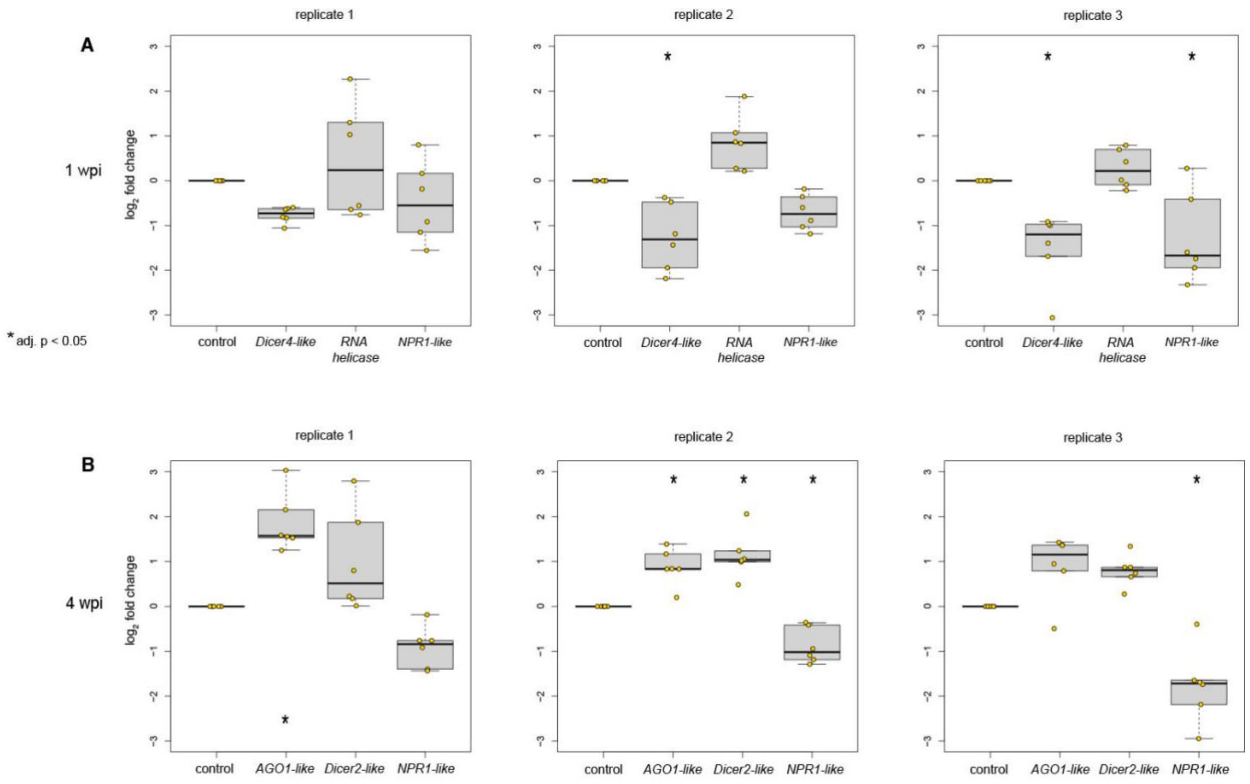

Supplemental Figure S2. qPCR validation of select genes in Russet Burbank plants at 1 and 4 wpi. (A) qPCR gene expression results from three biological replicates of PVY-infected Russet Burbank plants at 1 wpi. *Dicer4-like* (Soltu.DM.07G000040) exhibits decreases in expression in replicates 2 (-1.10 $\log_2$ fold change) and 3 (-1.35 $\log_2$ fold change), and a decreasing trend in replicate 1 (-0.75 $\log_2$ fold change), corroborating the transcriptomic analysis data. *NPR1-like* (Soltu.DM.07G014680) also exhibited a decrease in expression in replicate 3 (-0.98 $\log_2$ fold change) and downward trends in replicate 1 (-0.24 $\log_2$ fold change) and 2 (-0.65 $\log_2$ fold change), also matching the transcriptomic data. The *putative mitochondrial RNA helicase* (Soltu.DM.12G023910) increased in expression in the transcriptomic data, but was not found to increase by qPCR, though the replicates did exhibit a slight increasing trend of expression (0.90, 0.97, 0.32 $\log_2$ fold change). (**B)** qPCR gene expression results from three biological replicates of PVY-infected Russet Burbank plants at 4 wpi. *NPR1-like* exhibited lower expression in replicates 2 (-0.82 $\log_2$ fold change) and 3 (-1.56 $\log_2$ fold change) and trended lower in replicates 1 (-0.84 $\log_2$ fold change) compared to expression levels in mock-infected control plants. This data is similar to transcriptomic data. *AGO1-like* (Soltu.DM.03G019130) expression was greater in replicates 1 (1.99 $\log_2$ fold change) and 2 (0.92 $\log_2$ fold change), while *Dicer2-like* expression was greater in replicate 2 (1.23 $\log_2$ fold change) only and exhibited greater expression trends in replicates 1 (1.38 $\log_2$ fold change) and 3 (0.83 $\log_2$ fold change). Both genes displayed greater expression in the transcriptomic data. Statistical differences in gene expression between mock-infected and PVY-infected potato plants (n = 6) were performed using Wilcoxon ranked sum test, *p<0.05.

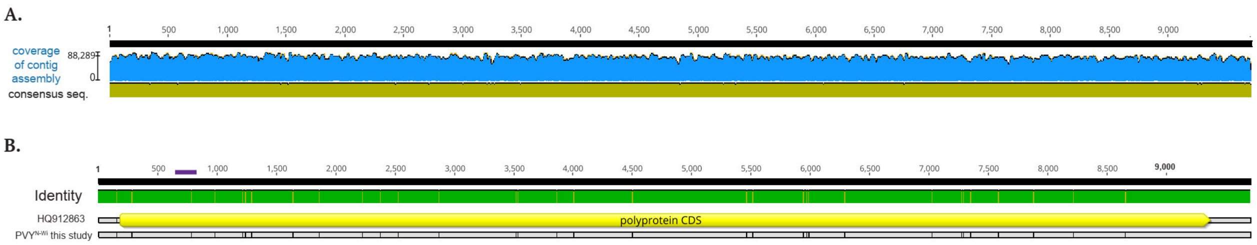

Supplemental Figure S3. PVY^{N-Wi} sequences from this study shares 99.7% nucleotide identity with PVY^{N-Wi} reference genome (HQ912863). (A) The PVY^{N-Wi} RNAseq consensus sequence in this study was generated by aligning 7,142,764 Illumina reads, from PVY-infected Russett Burkbank potato plant samples obtained at 4 weeks post-infection, to the PVY^{N-Wi} reference sequence (HQ12863).(B) The PVY^{N-Wi} consensus sequence in this study is 99.7% identical (9,666/9,698 nt) to the PVY^{N-Wi} reference sequence (HQ12863). The nucleotide identities are illustrated in green and the differences in nucleotide sequences are indicated by black vertical lines in the PVY^{N-Wi} seqence obtained in this study. The purple bar illustrates the region amplified by region amplified by qPCR (i.e., nt region #555-787). The images in this figure were generated using Geneious Prime 2021.2.2 (https://www.geneious.com).
